# Genetic variation in *CaTIFY4b* contributes to drought adaptation in chickpea

**DOI:** 10.1111/pbi.13840

**Published:** 2022-05-21

**Authors:** Rutwik Barmukh, Manish Roorkiwal, Vanika Garg, Aamir W. Khan, Liam German, Deepa Jaganathan, Annapurna Chitikineni, Jana Kholova, Himabindu Kudapa, Kaliamoorthy Sivasakthi, Srinivasan Samineni, Sandip M. Kale, Pooran M. Gaur, Someswar Rao Sagurthi, Yoselin Benitez‐Alfonso, Rajeev K. Varshney

**Affiliations:** ^1^ Centre of Excellence in Genomics and Systems Biology International Crops Research Institute for the Semi‐Arid Tropics (ICRISAT) Hyderabad India; ^2^ 28552 Department of Genetics Osmania University Hyderabad India; ^3^ Khalifa Center for Genetic Engineering and Biotechnology United Arab Emirates University Al‐Ain United Arab Emirates; ^4^ The UWA Institute of Agriculture The University of Western Australia Perth Western Australia Australia; ^5^ 4468 Centre for Plant Science School of Biology University of Leeds Leeds UK; ^6^ Crop Physiology and Modelling International Crops Research Institute for the Semi‐Arid Tropics (ICRISAT) Hyderabad India; ^7^ Crop Breeding International Crops Research Institute for the Semi‐Arid Tropics (ICRISAT) Hyderabad India; ^8^ 5673 Murdoch’s Centre for Crop & Food Innovation State Agricultural Biotechnology Centre Food Futures Institute Murdoch University Murdoch Western Australia Australia

**Keywords:** legumes, terminal drought, seed weight, root system architecture, transpiration efficiency, vigour

## Abstract

Chickpea production is vulnerable to drought stress. Identifying the genetic components underlying drought adaptation is crucial for enhancing chickpea productivity. Here, we present the fine mapping and characterization of ‘*QTL‐hotspot*’, a genomic region controlling chickpea growth with positive consequences on crop production under drought. We report that a non‐synonymous substitution in the transcription factor *CaTIFY4b* regulates seed weight and organ size in chickpea. Ectopic expression of CaTIFY4b in *Medicago truncatula* enhances root growth under water deficit. Our results suggest that allelic variation in ‘*QTL‐hotspot*’ improves pre‐anthesis water use, transpiration efficiency, root architecture and canopy development, enabling high‐yield performance under terminal drought conditions. Gene expression analysis indicated that *CaTIFY4b* may regulate organ size under water deficit by modulating the expression of *GRF‐INTERACTING FACTOR1* (*GIF1*), a transcriptional co‐activator of Growth‐Regulating Factors. Taken together, our study offers new insights into the role of *CaTIFY4b* and on diverse physiological and molecular mechanisms underpinning chickpea growth and production under specific drought scenarios.

## Introduction

Drought is one of the most critical abiotic stresses that confines crop yields under rainfed conditions. The majority of the agricultural production under rainfed farming depends on residual soil moisture during the growing season. Hence, drought can have a potential calamitous impact on crop yields (Varshney *et al*., 2021a). There is a rising need to develop and adopt new crop varieties for improving crop yields in drought‐prone areas across the world, especially in South Asia and sub‐Saharan Africa (Atlin *et al*., 2017; Varshney *et al*., 2021b).

Owing to its high‐yield potential and nutritional value, chickpea (*Cicer arietinum* L.) is one of the most important legume crops cultivated on an area of ~14.84 million hectares with an annual production of ~15.08 million tonnes (FAOSTAT, 2020). Conventional breeding strategies have been successful over the past few decades in developing chickpea varieties that are more productive under the prevalent drought scenarios (Hajjarpoor *et al*., 2018; Kashiwagi *et al*., 2006; Singh *et al*., 1996). However, these approaches alone are not enough to keep pace with the future food demand. The realization of crop improvement can be substantially accelerated if assisted by innovations in genomics technologies, which have enabled rapid identification of genetic variation underlying crop performance and improved the efficiency of breeding (Roorkiwal *et al*., 2020; Varshney *et al*., 2021c). Notably, recent studies have successfully isolated and characterized candidate genes underlying the quantitative trait loci (QTLs) for complex traits using fine mapping and positional cloning approaches (Li *et al*., 2019; Sánchez‐Pérez *et al*., 2019; Su *et al*., 2019; Uga *et al*., 2013; Wang *et al*., 2015).

Although crop yield is a consequence of many plant functions and their interaction with the environment (Cooper *et al*., 2021; Tardieu, 2012), it is the ultimate target of crop improvement (Kholova *et al*., 2021). Crop yield under drought is constitutively determined by seed weight and the size of lateral organs, including leaf and root (Varshney *et al*., 2021a). Thus far, very few QTLs responsible for seed weight and/or organ size have been cloned and characterized in chickpea (Basu *et al*., 2019), although many have been identified in the last decade (Barmukh *et al*., 2021; Roorkiwal *et al*., 2018; Sivasakthi *et al*., 2018; Varshney *et al*., 2014). The ‘*QTL‐hotspot*’ region containing major effect QTLs for 12 drought‐adaptive traits, including 100‐seed weight, which explained up to 58.20% phenotypic variation was identified on linkage group 4 of chickpea (Varshney *et al*., 2014). Genotyping‐by‐sequencing and bin mapping‐based approaches delimited this region from ca. 7.74‐Mb to a ~307‐kb interval (Jaganathan *et al*., 2015; Kale *et al*., 2015). However, the candidate gene(s) controlling seed weight and organ size underlying ‘*QTL‐hotspot*’ remain to be elucidated due to a lack of fine mapping and functional validation.

In the present study, we used homozygous recombinant lines to delimit and validate the ‘*QTL‐hotspot*’ region controlling seed weight and organ size in chickpea. We describe the successful and effective screening of the chickpea composite collection, which revealed an allelic variant within the nuclear‐located *CaTIFY4b* gene associated with seed weight. *Medicago truncatula* (a closely related model plant species of chickpea) transgenic plants with enhanced *CaTIFY4b* expression exhibited improved survival rate and root growth under water deficit. Phenotypic evaluation of chickpea homozygous lines linked *CaTIFY4b* to the regulation of key physiological parameters, such as pre‐anthesis water use, transpiration efficiency (TE) and canopy development, associated with drought adaptation. Expression analyses revealed molecular components in this mechanism, including the regulation of *GROWTH‐REGULATING FACTOR (GRF)‐INTERACTING FACTOR1* (*GIF1*), previously shown to control organ size in *Arabidopsis* (Kim and Kende, 2004; Liu *et al*., 2020). We discuss these novel insights into the physiological and molecular basis of drought adaptation regulated by *CaTIFY4b* in chickpea.

## Results

### Fine mapping of the ‘*QTL‐hotspot*’ region reveals putative candidates controlling seed weight and root architecture

To identify genes controlling seed weight in chickpea, we developed a set of introgression lines using a drought adapted line, ICC 4958 (100‐seed weight, 25.79 g ± 0.19 g), as a donor, and a chickpea landrace, ICC 1882 (100‐seed weight 14.68 g ± 0.17 g), as a recipient. We obtained one introgression line (ICCX‐110125‐P18) in BC_5_F_1_ generation, with the highest genome recovery (98.04%) (Table S1), which harboured the ‘*QTL‐hotspot*’ region on Ca4 from ICC 4958. For high‐resolution mapping of the ‘*QTL‐hotspot*’, we used 1911 BC_6_F_2_ plants derived from a cross between ICCX‐110125‐P18 and ICC 1882. Comprehensive analysis of the recombination events between the flanking Kompetitive Allele Specific PCR (KASP) markers, CKAM2210 and CKAM2182, revealed 22 types of genotype classes (Table S2 and Figure S1). A total of 284 plants obtained from 19 BC_6_F_2:3_ families were genotyped initially with six KASP markers, followed by 18 newly developed KASP markers to identify more recombinations within the target region (Figure S2). To clarify the genetic effects of the ‘*QTL‐hotspot*’ on 100‐seed weight, we analyzed BC_6_F_3_ plants homozygous for ICC 1882 (ICC 1882‐homo) and ICC 4958 (ICC 4958‐homo) alleles. The mean 100‐seed weight of ICC 4958‐homo (20.88 ± 0.77 g) was significantly larger than that of ICC 1882‐homo (13.27 ± 0.26 g) at *P* < 0.001 (Figure S3). Therefore, it was confirmed that the ICC 4958 allele of ‘*QTL‐hotspot*’ contributed to increased seed weight in chickpea.

**Figure 1:**
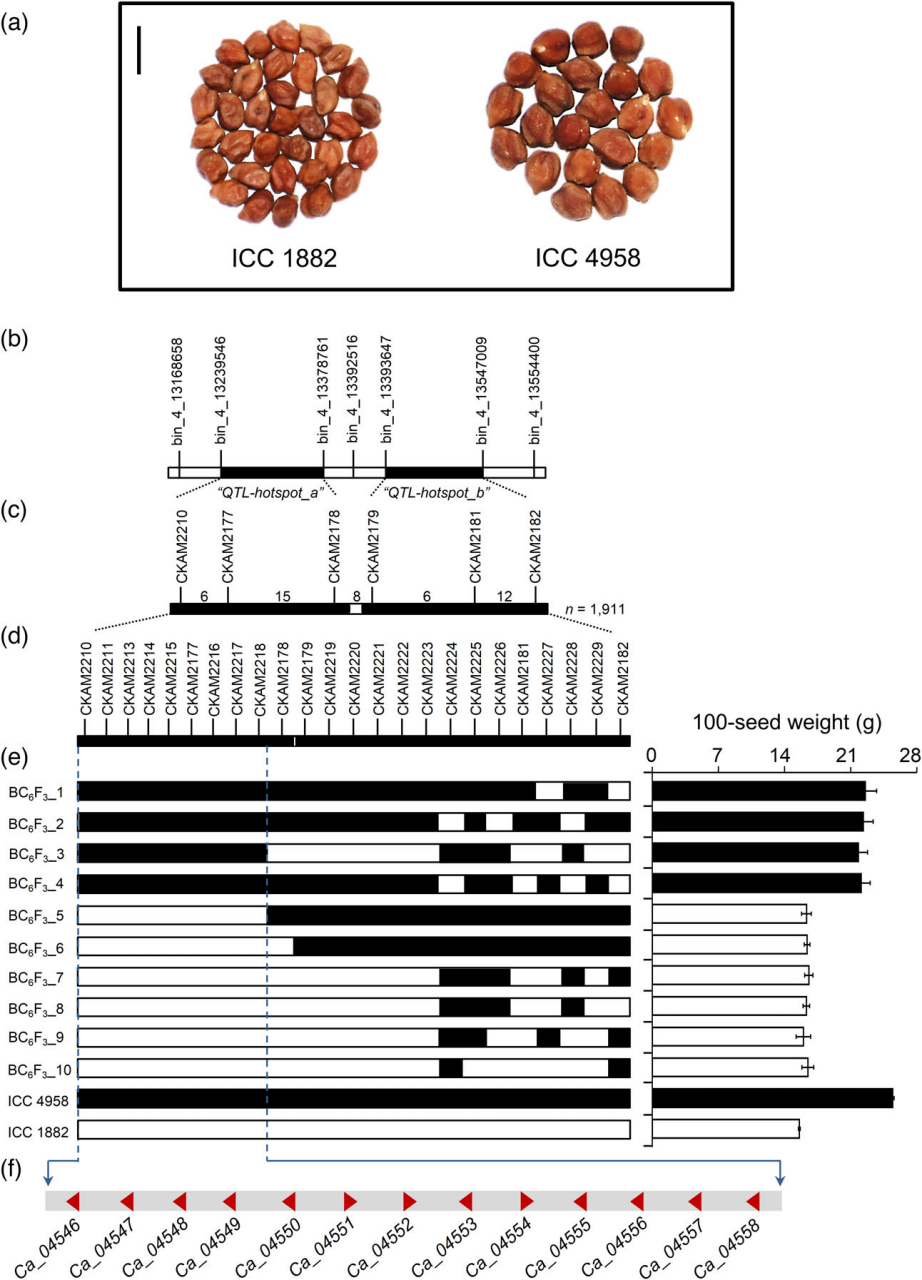
Fine mapping of the ‘*QTL‐hotspot*’ region. (a) Seed phenotypes of parental lines (ICC 1882 and ICC 4958). Scale bar, 1 cm. (b) Refinement of the ‘*QTL‐hotspot*’ region on chickpea chromosome Ca4 into two sub‐regions (filled bars), viz. ‘*QTL‐hotspot_a*’ (139.22‐kb; 0.23 cM) and ‘*QTL‐hotspot_b*’ (153.36‐kb; 0.22 cM), reported by Kale *et al*. (2015). (c) Linkage map of ‘*QTL‐hotspot_a*’ and ‘*QTL‐hotspot_b*’ sub‐regions developed using 1911 BC_6_F_2_ plants. The number of recombinants between the adjacent markers is represented above the linkage map. (d) High‐resolution linkage map containing 24 KASP markers developed based on SNPs present within ‘*QTL‐hotspot_a*’ and ‘*QTL‐hotspot_b*’ sub‐regions using BC_6_F_3_ plants. (e) Progeny testing of fixed recombinant plants (BC_6_F_4_) delimited the ‘*QTL‐hotspot*’ region controlling 100‐seed weight and root architecture to ~113.04‐kb region on ‘*QTL‐hotspot_a*’ between the markers CKAM2210 and CKAM2218. The 100‐seed weight (mean ± SE) of first four recombinant families (BC_6_F_3__1 to BC_6_F_3__4) was higher than that of remaining six recombinant families (BC_6_F_3__5 to BC_6_F_3__10) and ICC 1882. Filled and open bars indicate homozygous chromosomal segments for ICC 4958 and ICC 1882, respectively. BC_6_F_3__1 to BC_6_F_3__10, 10 recombinant families. (f) Genes annotated in the delimited the ‘*QTL‐hotspot*’ region using the CDC Frontier reference genome. Gene annotations are provided in Table S4.

Further, we conducted fine mapping of the ‘*QTL‐hotspot*’ region. To map the 100‐seed weight QTL as a single locus, we evaluated the homozygous lines under rainfed field conditions. A progeny test of homozygous segregates classified 50 BC_6_F_4_ plants into two groups that exhibited either high or low 100‐seed weight. A total of 19 plants belonging to four families (BC_6_F_3__1 to BC_6_F_3__4) showed a high 100‐seed weight (16.88–20.23 g); whereas, 31 plants belonging to the remaining six families (BC_6_F_3__5 to BC_6_F_3__10) displayed a low 100‐seed weight (14.12–14.95 g) (Table S3). These groups resembled genotype classes that are homozygous for the ICC 4958 allele of ‘*QTL‐hotspot*’ (ICC 1882 + Qh) and ICC 1882 allele of ‘*QTL‐hotspot*’ (ICC 1882 ↔ Qh), respectively. Further, we investigated the effects of the ‘*QTL‐hotspot*’ region on root growth and architecture of plants at 35 days after sowing (DAS). We observed a significant increase in root length density (by 57.0%), root dry weight (by 128.1%), root surface area (by 63.2%) and root volume (by 73.3%) of ICC 1882 + Qh plants compared with ICC 1882 plants (Figure S4a–f). This increase in root parameters was mainly attributed to major phenotypic differences in 0–30 cm depth interval (Figure S4g–i). Taken together, these results demonstrate that the ‘*QTL‐hotspot*’ region controlling seed weight and root system architecture was narrowed down to ~113.04‐kb interval between the markers CKAM 2210 and CKAM 2218 (Figure 1a–e). This region contained 13 putative genes, based on the annotation information of *Cicer arietinum* CDC Frontier genome (Varshney *et al*., 2013a) (Figure 1f and Table S4).

### Analysis of the chickpea composite collection uncovers natural variation in genes underlying the ‘*QTL‐hotspot*’ region

**Figure 2:**
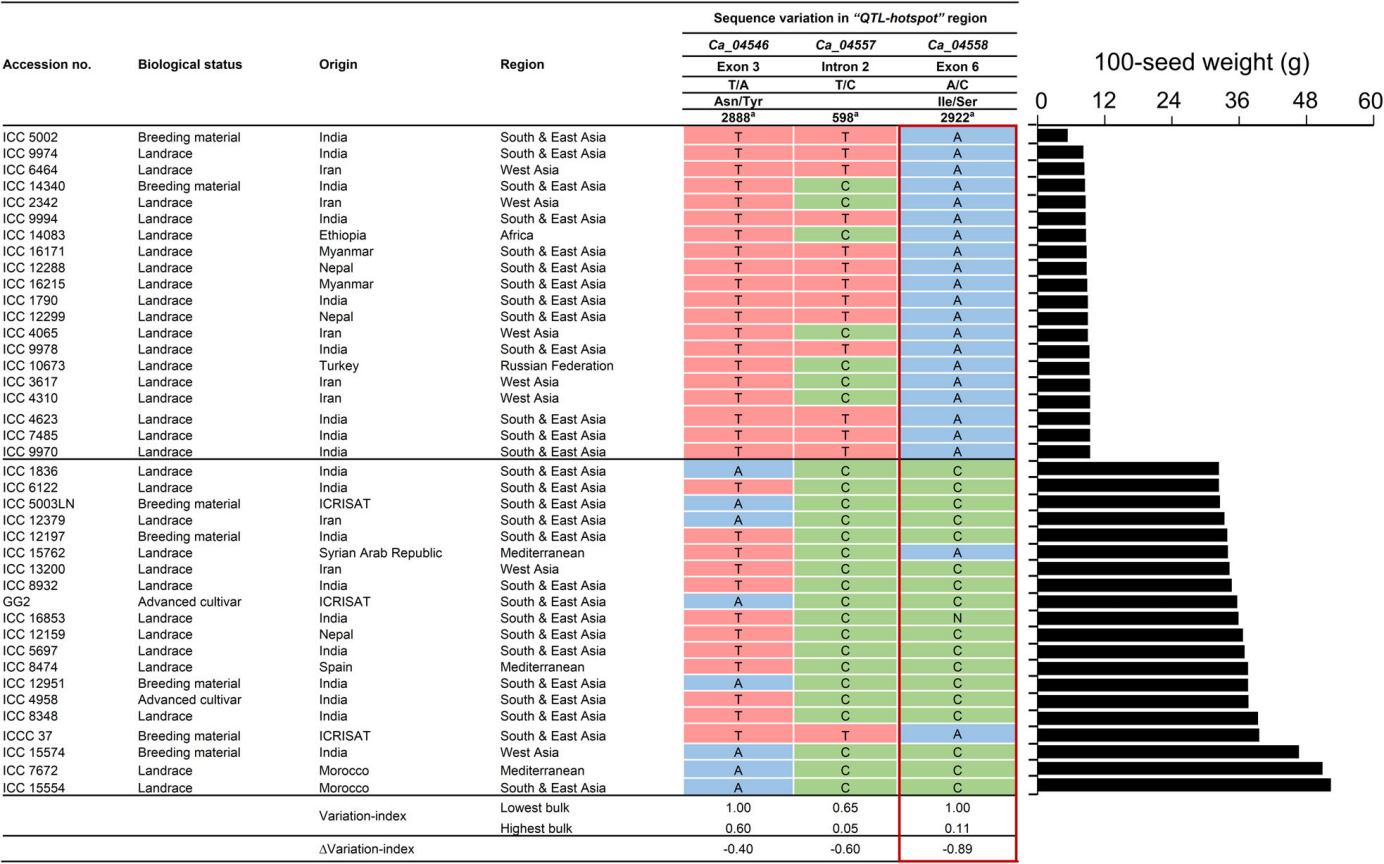
Sequence polymorphism in the genes underlying the ‘*QTL‐hotspot*’ region and natural variation in 100‐seed weight amongst chickpea germplasm accessions. Top, 20 extreme germplasm accessions with low 100‐seed weight classified as lowest bulk. Bottom, 20 extreme germplasm accessions with high 100‐seed weight classified as highest bulk. The three SNP mutations present within three candidate genes underlying ‘*QTL‐hotspot*’, which are closely associated with the variation in 100‐seed weight of the extreme bulks are enlisted. ICC, ICRISAT Chickpea assigned to ICRISAT germplasm accessions; N, not available. ^a^SNP position from start of the reference genome sequence.

To further narrow the distance of the ‘*QTL‐hotspot*’ region, we scrutinized 1712 desi accessions from the chickpea composite collection that showed large phenotypic variation for 100‐seed weight (5.30–52.30 g). We selected 20 accessions with extreme phenotypic values and categorized them into ‘lowest’ and ‘highest’ bulks to enhance the chances of identifying allelic diversity whilst maintaining a limited number of accessions. A significant difference was observed between the phenotypic data of two extreme bulks (at *P* < 0.001). Of the 13 putative genes (Table S4), single‐nucleotide variations were detected in the coding or non‐coding regions of three genes, including a 1 bp non‐synonymous substitution in *Ca_04546* (T2888A), a point mutation in the intron of *Ca_04557* (T598C) and a 1 bp non‐synonymous substitution in *Ca_04558* (A2922C) (Figure 2). The ΔVariation‐index values ranged from −0.40 to −0.89, suggesting *Ca_04558* with a ΔVariation‐index value of −0.89 as the strongest candidate. Supporting this result, the diagnostic marker (CKAM2217) developed for the SNP (A2922C) in *Ca_04558* gene differentiated the homozygous lines based on 100‐seed weight (Table S3). Collectively, these results implied that a point mutation in the *Ca_04558* gene may account for the differences in seed weight between ICC 4958 and ICC 1882, although contributions from the other two genes cannot be ignored.

**Figure 3:**
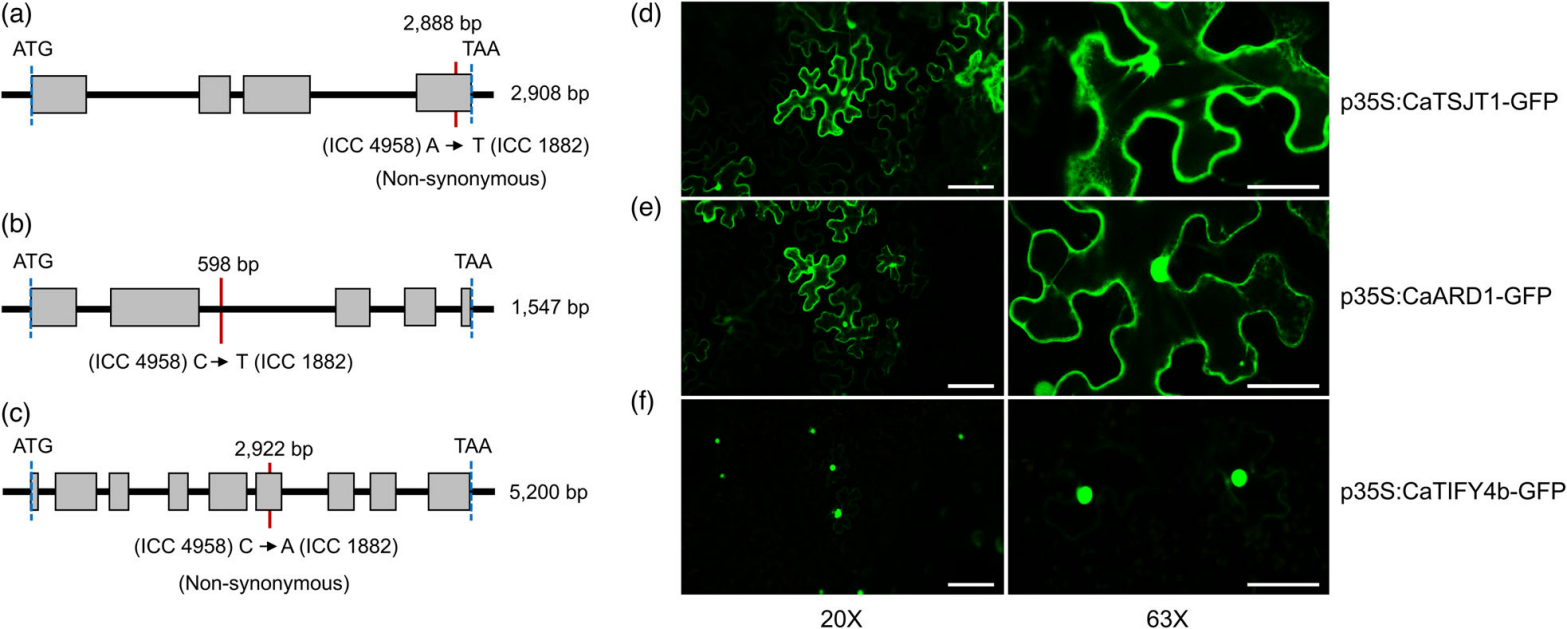
Structural variation in the prioritized genes and subcellular localization of their fusion proteins. (a) *CaTSJT1*, (b) *CaARD1* and (c) *CaTIFY4b* gene structure based on the annotation of *Ca_04546*, *Ca_04557* and *Ca_04558*, respectively. Grey rectangles, open reading frame; red line, single‐nucleotide substitution in ICC 4958 relative to ICC 1882; blue dashed line, position of start and end codons of the gene. (d–f) Subcellular localization of (d) CaTSJT1‐GFP, (e) CaARD1‐GFP and (f) CaTIFY4b‐GFP fusion proteins after transient expression in *N*. *benthamiana* leaves, driven by the *35S* promoter. Left, images at 20× magnification (scale bar, 100 µm); right, images at 63× magnification (scale bar, 50 µm).

A gene ontology (GO) analysis predicted the three prioritized genes to be involved in asparagine biosynthesis, methionine metabolic process and leaf development (Table S4). The *Ca_04546* gene (named *CaTSJT1*) displays a non‐synonymous substitution within the fourth exon (Figure 3a) and CaTSJT1 protein shares 91.20% identity (http://www.ncbi.nlm.nih.gov/BLAST) with the stem‐specific protein TSJT1 from *M. truncatula*. Based on database searches, we identified *CaTSJT1* homologs from different legume species (Figure S5), although the function of this protein remains largely unknown. The *Ca_04557* gene (named *CaARD1*) contains a point mutation in the second intron (Figure 3b). The CaARD1 protein is 90.37% identical to *M. truncatula* 1,2 dihydroxy‐3‐keto‐5‐methylthiopentene dioxygenase 1 (ARD1), a protein known to regulate ethylene and polyamine biosynthesis (Sauter *et al*., 2005). Finally, the third and most promising candidate *Ca_04558* (named *CaTIFY4b*) contains a 1 bp non‐synonymous substitution within exon 6 (Figure 3c) and is predicted to encode a 339‐residue polypeptide (~36.97 kDa) containing a TIFY‐like domain that characterize a family of transcription factors (Vanholme *et al*., 2007). Database searches identified CaTIFY4b homologous sequences from diverse legume species (Figure S6). A BLASTP analysis indicated that CaTIFY4b shares a high identity (82.93%) with BIG SEEDS1 (BS1) from alfalfa and *M. truncatula* (Ge *et al*., 2016). Analysis using the ‘PredictProtein’ database (Yachdav *et al*., 2014) revealed that the TIFY domain forms a helical structure and is deeply buried inside the protein core. Importantly, the 1 bp non‐synonymous substitution (I149S) caused by the SNP mutation in *CaTIFY4b* led to alterations in the highly conserved TIFY motif (Figure S7).

### Subcellular localization and transgenic expression suggest that CaTIFY4b acts in the nucleus to regulate root growth under water deficit

To determine the potential function of prioritized genes underlying the ‘*QTL‐hotspot*’ region, we made Green Fluorescent Protein (GFP)‐tagged constructs that were transiently expressed in *Nicotiana benthamiana* leaves using agroinfiltration. Analysis of protein sequences and the literature identified the C‐terminal as the correct location for these fusions. Confocal imaging of transformed leaf epidermal cells revealed the subcellular localization of these proteins. Both CaTSJT1‐GFP and CaARD1‐GFP fusion proteins appeared to accumulate in the nucleus, endoplasmic reticulum, plasma membrane and, perhaps, the cytoplasm (Figure 3d,e). The expression of CaTIFY4b‐GFP fusion protein was mostly restricted to the nucleus, consistent with a putative function as a transcription factor (Figure 3f). Importantly, the fluorescence signal suggested that protein fusions were successful and did not affect protein stability.

**Figure 4:**
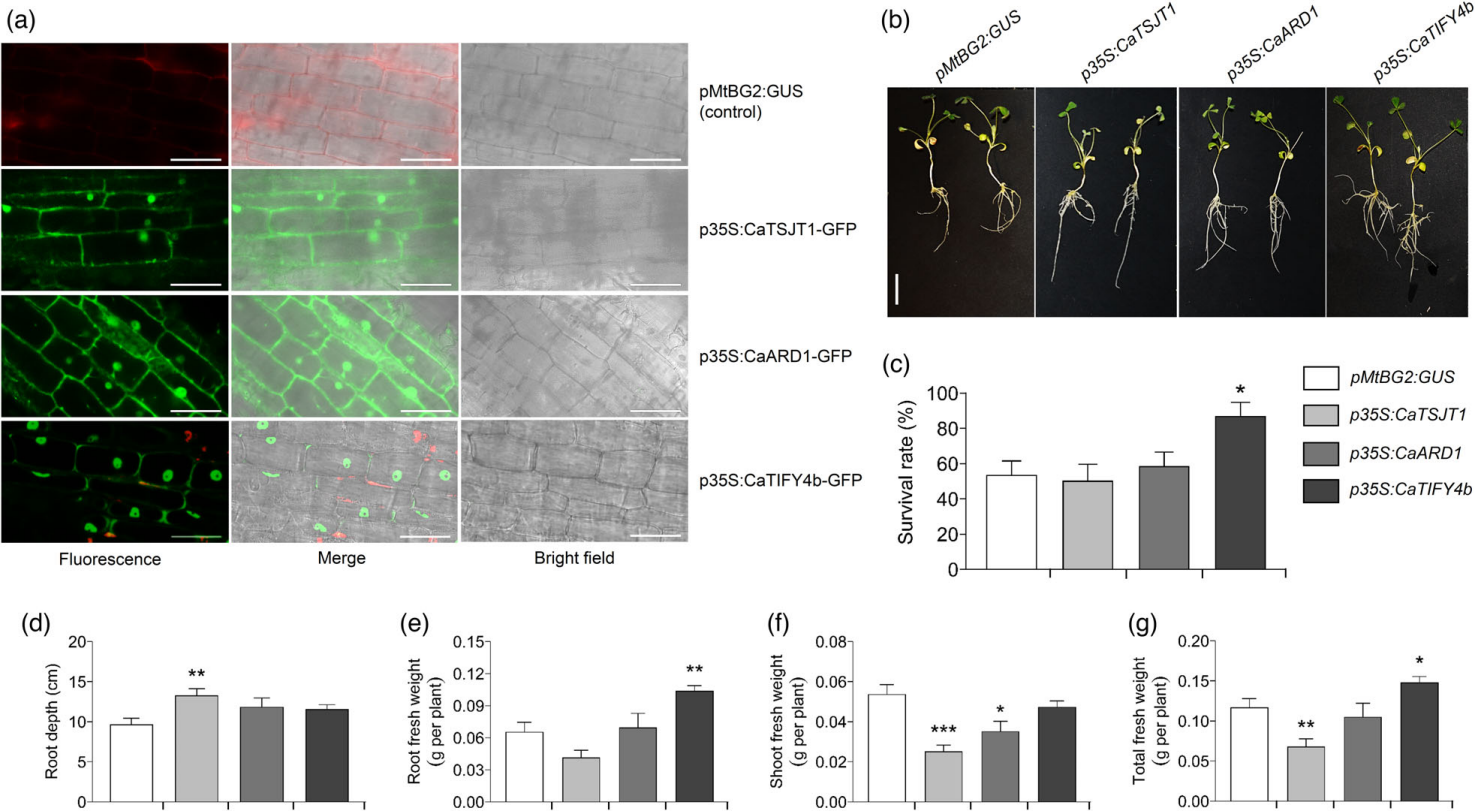
Functional characterization of prioritized genes in *M*. *truncatula*. (a) The pMtBG2:GUS (control), p35S:CaTSJT1‐GFP, p35S:CaARD1‐GFP and p35S:CaTIFY4b‐GFP constructs were ectopically expressed in *M*. *truncatula* hairy roots. Images were taken using confocal microscopy with the GFP (488 nm), RFP (550 nm) and bright field channels. Left, fluorescence in the GFP channel; centre, merged fluorescence and bright field images; right, bright field channel. Scale bar, 50 µm. (b) Representative pictures of *p35S:CaTSJT1*, *p35S:CaARD1* and *p35S:CaTIFY4b* transgenic plants compared to control (*pMtBG2:GUS*). Photographs were captured before transfer to soil. Scale bar, 2 cm. (c) Statistical data for survival rate of the *pMtBG2:GUS* (control), *p35S:CaTSJT1*, *p35S:CaARD1* and *p35S:CaTIFY4b* transgenic plants when exposed to drought stress. (d–g) Statistical comparisons of (d) root depth, (e) root fresh weight, (f) shoot fresh weight and (g) total fresh weight that were used to quantify differences between *pMtBG2:GUS*, *p35S:CaTSJT1*, *p35S:CaARD1* and *p35S:CaTIFY4b* plants subjected to water deficit (for 8 days). Error bars, SE; statistical significance was determined by a two‐sided *t*‐test: *P < 0.05, **P < 0.01.

Next, we expressed the prioritized genes in *M. truncatula* (A17) using *Agrobacterium rhizogenes*‐mediated hairy root transformation. *M. truncatula* provides a suitable heterologous model system to rapidly study the potential function of chickpea genes in the drought response (Nayak *et al*., 2010). Transgenic roots ectopically expressing *CaTSJT1*, *CaARD1* and *CaTIFY4b* genes in the wild‐type *M. truncatula* were generated and the localization of the fusion proteins was analysed as a control of successful transformation. A construct expressing GUS (β‐glucuronidase) under the *MtBG2* promoter (Gaudioso‐Pedraza *et al*., 2018) was used as a negative control for transformation. The localization of these proteins in *M. truncatula* roots resembled that observed in tobacco leaves (Figure 3d–f). For instance, the CaTSJT1‐GFP and CaARD1‐GFP fusion proteins localized to the nucleus with a diffuse pattern in other cellular localizations (likely in the secretory pathway, plasma membrane and cytosol) (Figure 4a). The CaTIFY4b‐GFP protein displayed a clear nuclear localization with some residual signal in the cell periphery (Figure 4a).

To phenotypically characterize protein function, we analysed the survival rate, root and shoot traits in several independent plants with transgenic roots that were grown on soil under well‐watered (WW) and water stress (WS) conditions (Figure 4b). The transgenic plants with enhanced *CaTIFY4b* gene expression in the hairy roots exhibited a significantly increased survival rate relative to control (*pMtBG2:GUS*) plants under water deficit conditions (Figure 4c). Compared to *pMtBG2:GUS* plants, the *p35S:CaTSJT1*, *p35S:CaARD1* and *p35S:CaTIFY4b* plants displayed higher root depth, although differences were only significant for *p35S:CaTSJT1* under WS conditions (Figure 4b,d). Root mass increased significantly in *p35S:CaTIFY4b* plants under water deficit, whereas it remained unaffected in *p35S:CaTSJT1* and *p35S:CaARD1* plants (Figure 4e). Interestingly, a substantial reduction in shoot and total plant biomass under WS was observed for *p35S:CaTSJT1* and *p35S:CaARD1* plants, whereas, *p35S:CaTIFY4b* plants were able to maintain shoot mass and significantly increased total plant biomass (Figure 4f,g). No major phenotypic changes were detected in *p35S:CaARD1* and *p35S:CaTIFY4b* plants compared to *pMtBG2:GUS* plants, under WW conditions (Figure S8a–d). The *p35S:CaTSJT1* plants, on the other hand, showed a significant decrease in root mass, shoot mass and total plant biomass in WW experiments.

The results, so far, suggest that ectopic expression of *CaTIFY4b* is sufficient to improve root growth without negatively affecting shoot growth under water deficit. We further compared the root growth and architecture traits of *p35S:CaTIFY4b* and *pMtBG2:GUS* hairy root plants grown on Fahraeus Plant (FP) medium plates. Notably, in these conditions, *p35S:CaTIFY4b* plants had significantly higher root depth (116.1%), surface area (145.4%) and growth rate (87.2%) than those of their control‐transformed counterparts (Figure S8e–g). Taken together, these results demonstrate that overexpression of CaTIFY4b in *M*. *truncatula* roots improves root growth and architecture affecting plant response to water deficit.

### Allelic variation in the ‘*QTL‐hotspot*’ region is associated with seed yield, water uptake patterns, TE and canopy development

**Figure 5:**
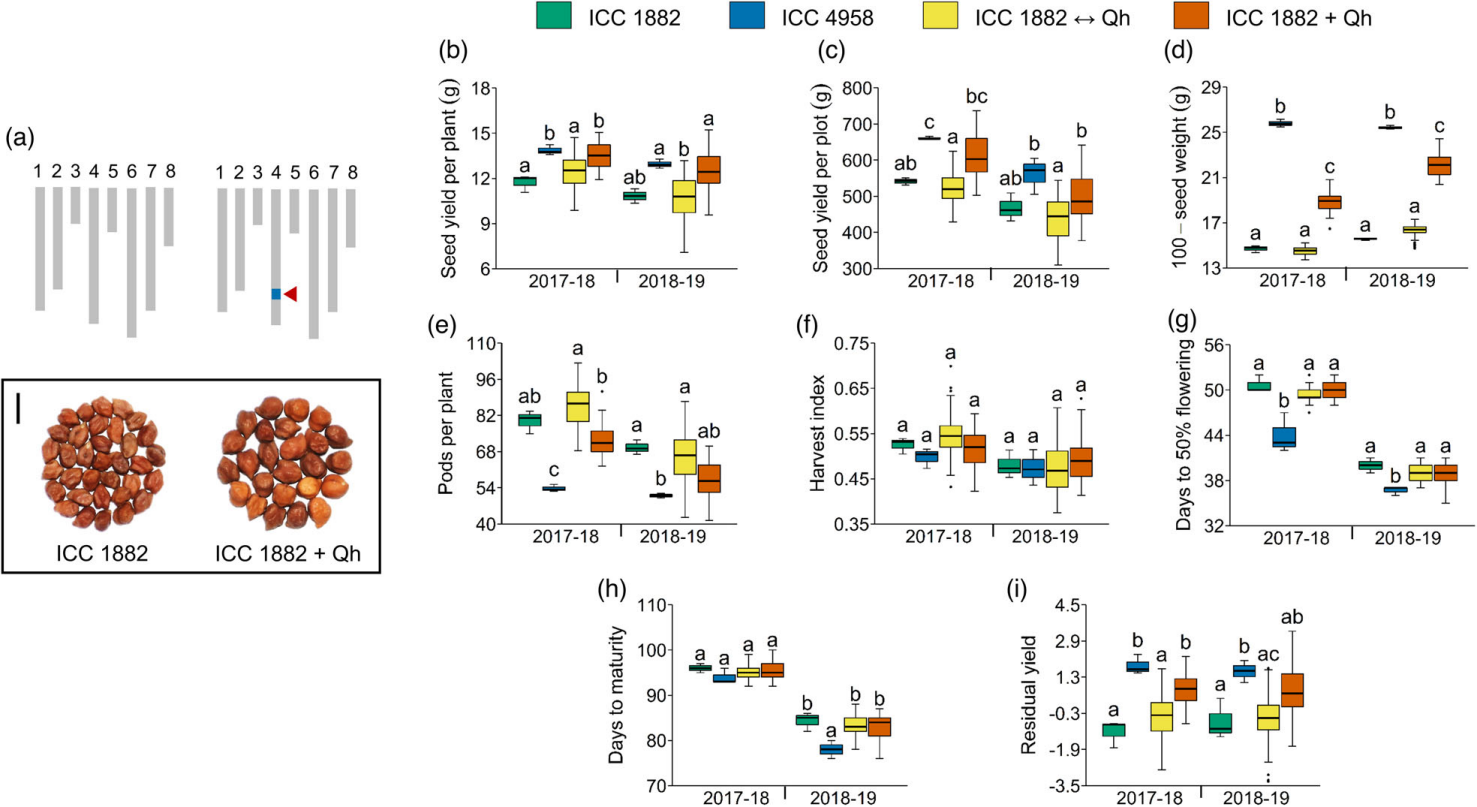
Effect of the ‘*QTL‐hotspot*’ region on seed yield, yield components and phenology traits evaluated under rainfed field conditions. (a) Graphical genotypes (chromosome Ca1‐8) and seed phenotype of ICC 1882 and ICC 1882 + Qh plants. Grey rectangles, homozygous regions from ICC 1882; blue rectangle, homozygous region from ICC 4958; red arrowhead, position of ‘*QTL‐hotspot*’. Scale bar, 0.8 cm. (b–i) Comparison of ICC 1882, ICC 4958, ICC 1882 ↔ Qh and ICC 1882 + Qh plants for (b) seed yield per plant, (c) seed yield per plot, (d) 100‐seed weight, (e) pods per plant, (f) harvest index, (g) days to 50% flowering, (h) days to maturity and (i) residual yield. The year in which phenotypic traits were evaluated is shown on the *x*‐axis. For the box plots, boxes denote the 25th–75th percentile, whiskers denote the full data range and the centre lines denote medians. The alphabets above the boxes (a, b, c) in panels (b–i) designate statistical significance between the groups of genotypes computed using Tukey’s test (*P* < 0.05).

To investigate the physiological basis of drought adaptation regulated by the ‘*QTL‐hotspot*’ region (with *CaTIFY4b* as a major contributor) in chickpea, we evaluated the homozygous recombinant lines at the field, rain‐out shelter, glasshouse and LeasyScan platforms (Tables S5 and S6). Here, ICC 1882 + Qh plants possessed the ICC 4958 allele of the ‘*QTL‐hotspot*’; whereas, ICC 1882 ↔ Qh plants contained the ICC 1882 allele of the ‘*QTL‐hotspot*’ (Figure 5a and Table S3). Under rainfed field conditions, most of the yield, yield components and phenology traits displayed a significant variation in genotype (G) and genotype × environment (year) (G × E) interactions (Tables S7 and S8). We found that seed yield per plant of ICC 1882 + Qh plants increased by 15.0% (2017–18) and 15.2% (2018–19) compared to ICC 1882 plants, and this difference was significant for the 2017–18 experiment (Figure 5b,c). Although the 100‐seed weight of ICC 1882 + Qh plants showed a major increase over ICC 1882 plants (Figure 5d); the number of pods per plant, harvest index, days to 50% flowering and days to maturity did not differ significantly in ICC 1882 + Qh than in ICC 1882 plants (Figure 5e–h). The residual yield, used as a proxy for drought adaptation, of ICC 1882 + Qh plants was higher than that of ICC 1882 plants (Figure 5i).

We evaluated the homozygous lines under rain‐out shelter environments to identify the effect of ‘*QTL‐hotspot*’ on water uptake patterns. Under WS conditions, the pre‐anthesis water use of ICC 1882 + Qh plants was higher by 20.3% (2017–18 WS) and 13.3% (2018–19 WS) than that of ICC 1882 plants, and this difference was substantial for the 2017–18 experiment (Figure S9a). An increase in TE by 17.9% (2017–18 WS) and 5.2% (2018–19 WS) in ICC 1882 + Qh plants was observed compared to that of ICC 1882 plants, which was significant for the 2017–18 trial (Figure S9a). Under WW conditions, the pre‐anthesis water use of ICC 1882 + Qh plants showed a considerable increase over ICC 1882 plants during 2017–18 WS, but no major differences were observed in post‐anthesis water use, cumulative water use and TE (Figure S9b).

At the pod‐filling stage, root depth and root dry weight displayed a significant genotype × treatment (G × T) interaction at *P* < 0.05 (Table S8), suggesting a strong water deficit influence on these traits. Under WS and WW conditions, root parameters did not display significant differences between ICC 1882 + Qh and ICC 1882 plants (Figure S10a,b), implying that the ‘*QTL‐hotspot*’ mainly regulates root growth and architecture at the vegetative stage. Further, we evaluated the canopy development parameters at LeasyScan platform and rainfed field conditions. The 3D‐leaf area of ICC 1882 + Qh plants was remarkably higher by 101.8% (2017–18) and 51.4% (2018–19) than that of ICC 1882 plants, whereas the projected leaf area was significantly greater by 119.1% (2017–18) and 51.7% (2018–19) in ICC 1882 + Qh plants compared to ICC 1882 plants (Figure S11a–c). The leaf area index (a measure of canopy growth), shoot digital biomass and specific leaf area of ICC 1882 + Qh plants were significantly higher than that of ICC 1882 plants (Figure S11d–f). Under field conditions, shoot biomass of ICC 1882 + Qh plants was higher by 17.3% (2017–18) and 12.2% (2018–19) than that of ICC 1882 plants (Figure S11g). The ICC 1882 + Qh plants displayed a substantial increase in plant height (by 9.0–9.7%) (Figure S11h) and plant vigour (by 63.8–67.8%), relative to ICC 1882 plants (Figure S11i). Moreover, the leaves of ICC 1882 + Qh plants responded more abruptly to an increase in leaf area than those of ICC 1882 plants (Figure S12).

The experimental trials in which a significant variation in drought‐adaptive traits was observed between ICC 1882 + Qh and ICC 1882 plants were considered for establishing trait relationships. Under field conditions (2017–18), seed yield per plant improved with an increase in residual yield per plant, which was closely related to 100‐seed weight (Figure S13). To identify the impact of water extraction patterns on yield and its components, we compared seed yield per plant from the field (2017–18) with traits from rain‐out shelter lysimeters (2017–18 WS). Seed yield per plant was closely associated with pre‐anthesis water use, whereas an increase in pod number was linked to post‐anthesis water use (Figure S14a). The accumulation of shoot biomass favoured an increase in TE, which, in turn, was positively associated with 100‐seed weight (Figure S14a). A significant negative correlation was observed between water extracted in the pre‐anthesis and post‐anthesis stage (Figure S15), suggesting that larger plants with more water extraction early in the crop cycle tended to have less water available in the soil profile during the pod‐filling stage. Under WW conditions, an increase in cumulative water uptake favoured seed yield, whilst differences in 100‐seed weight were predominantly related to pre‐anthesis water use and TE (Figure S14b).

To identify the effects of canopy development on seed yield, we combined canopy development traits captured by the LeasyScan platform (2017–18) with seed yield data generated at rain‐out shelter lysimeters (2017–18 WW). Seed yield was closely related to early vigour‐related traits including plant height, digital biomass and 3D‐leaf area (Figure S16a). An increase in water extracted during early growth stages under water deficit was tightly linked to plant height and early vigour‐related traits (Figure S16b). Further, we compared the root traits evaluated at the vegetative stage with water uptake patterns from glasshouse lysimeters (2018–19 WS). Pre‐anthesis water use was positively correlated with root length density, root dry weight and root surface area (Figure S17a). Notably, the root traits evaluated during the pod‐filling stage were closely related to pre‐anthesis water use (Figure S17b).

The above results suggest that the ICC 4958 allele of the ‘*QTL‐hotspot*’ improves seed yield, pre‐anthesis water use, TE and canopy growth. Since *CaTIFY4b* is a major contributor of the ‘*QTL‐hotspot*’, we propose that ICC 1882 + Qh effects are caused by the presence of the *CaTIFY4b*‐ICC 4958 allele in the ICC 1882 background. Our data indicate that the increase in seed yield, under a particular drought scenario, is likely facilitated by the *CaTIFY4b*‐ICC 4958 allele and is a consequence of enhanced pre‐anthesis water use linked to vigorous plant growth and higher TE.

### Genome‐wide transcriptome and gene expression analyses provide insights into the molecular mechanisms underlying seed weight and leaf size under water deficit

**Figure 6:**
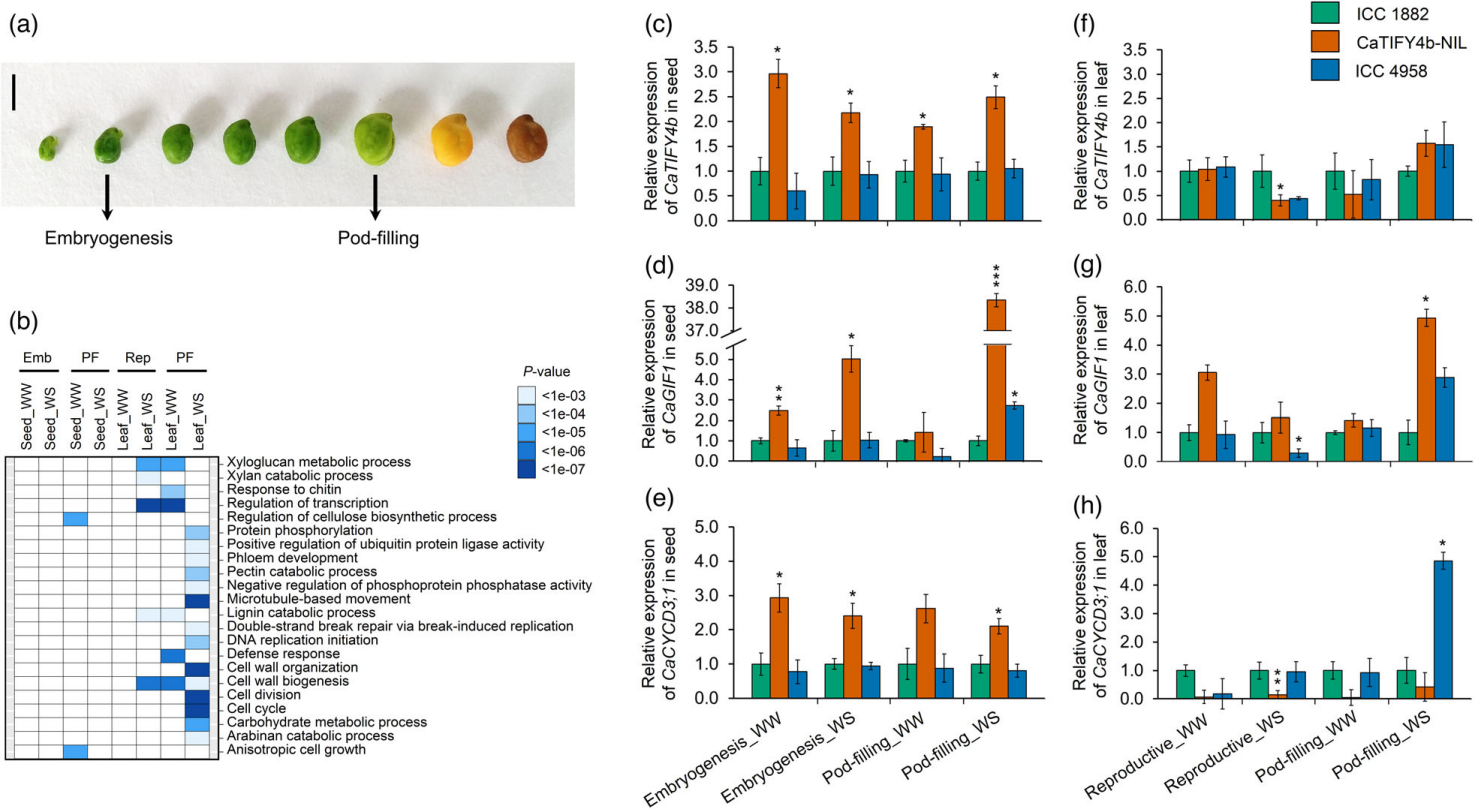
Differential gene expression at diverse stages of seed and leaf development in well‐watered and water stress conditions. (a) Phenotype of chickpea (ICC 4958) seed at different stages of development (embryogenesis and pod‐filling are arrowed). (b) Gene ontology (GO) enrichment terms (biological process) for seed and leaf samples at different stages of development and water treatments is shown. Emb, embryogenesis; PF, pod‐filling; Rep, reproductive. (c–e) The relative expression levels of (c) *CaTIFY4b*, (d) *CaGIF1* and (e) *CaCYCD3;1* at the embryogenesis and pod‐filling stages under WW and WS conditions, in the seeds of ICC 1882, CaTIFY4b‐NIL and ICC 4958 were detected by qRT‐PCR (*n* = 3). (f–h) The relative expression levels of (f) *CaTIFY4b*, (g) *CaGIF1* and (h) *CaCYCD3;1* at the reproductive and pod‐filling stage under WW and WS conditions, in the leaves of ICC 1882, CaTIFY4b‐NIL and ICC 4958 were detected by qRT‐PCR (*n* = 3). The qRT‐PCR data were normalized with *GAPDH*. *P < 0.05, Student’s *t*‐test.

To better understand the mechanisms underlying the regulation of seed and leaf size, we compared the transcriptomes of ICC 4958 and ICC 1882 under WW and WS conditions, by RNA‐sequencing (RNA‐seq). Two stages of seed development emphasizing key events taking place within the seeds, for example, embryo development (embryogenesis) and storage reserve accumulation (pod‐filling), were scrutinized (Figure 6a). Hierarchical clustering, global correlation and principal component analysis (PCA) revealed that the seed and leaf samples were separated from each other irrespective of the genotypes and water treatments (Figure S18). A total of 1074 differentially expressed genes (DEGs) were identified (adj. *P* < 0.05) at different stages of seed development, of which 62.0% (666 genes) were up‐regulated and 38.0% (408 genes) were down‐regulated in ICC 4958 relative to ICC 1882. In leaf samples, 4703 DEGs were detected (adj. *P* < 0.05) at different stages, of which 46.8% (2199 genes) were up‐regulated and 53.2% (2504 genes) were down‐regulated in ICC 4958 compared to ICC 1882.

The GO terms, regulation of cellulose biosynthetic process and anisotropic cell growth were highly enriched at the pod‐filling stage of seed development under WW conditions (Figure 6b). Various cell proliferation‐related terms, such as cell division, cell cycle and DNA replication initiation, and processes related to carbohydrate metabolism, pectin and arabinan catabolism, were substantially enriched in up‐regulated genes in the leaf, particularly at the pod‐filling stage under water deficit (Figure 6b). To determine the metabolic pathways regulating the differences in seed and leaf size, we analysed the DEGs based on the MapMan bins. In the seed, pathways that could influence energy requirements of the actively dividing cells (e.g., carbohydrate metabolism, lipid metabolism and secondary metabolism) were affected at the embryogenesis stage (Table S9). In the leaf, genes involved in cell cycle organization, chromatin organization and protein modification, amongst others, exhibited higher expression in ICC 4958 at the pod‐filling stage under water deficit (Table S9).

Search results of the chickpea Gene Expression Atlas (Kudapa *et al*., 2018) revealed that *CaTIFY4b* is expressed in all major tissues, with the highest expression in the immature seed during the reproductive stage (Figure S19). We used the RNA‐seq data to query the expression profile of *CaTIFY4b* downstream target genes. The *CaTIFY4b* homologs, *BS1* and *PEAPOD (PPD)*, are known to regulate seed and/or organ size in *Medicago* (Ge *et al*., 2016) and *Arabidopsis* (White, 2006), respectively. A large number of core cell cycle genes including the A‐, B‐ and D‐type cyclins (CYC) and genes governing cell proliferation activity such as *GIF1* and *GRF5* (*GROWTH REGULATING FACTOR5)* were differentially regulated in the *mtbs1‐1* plants (Ge *et al*., 2016). In chickpea seeds, the expression of *GIF1* was found to be lower at the embryogenesis stage (WS) and higher at the pod‐filling stage (WS) in ICC 4958 relative to ICC 1882 (Figure S20a). The *CYCA2;1*, *CYCB1;1* and *CYCD3;1* genes showed lower expression at the embryogenesis stage (WS) and higher expression at the pod‐filling stage (WS) in ICC 4958 seed when compared to ICC 1882 (Figure S20a). Furthermore, the transcriptional activity of *GIF1* and *GRF5* showed a marked increase in ICC 4958 leaves at the pod‐filling stage under water deficit. The expression of genes encoding some of the bonafide components of cell cycle machinery was lower in ICC 4958 at the reproductive stage and higher at the pod‐filling stage under WS conditions (Figure S20b).

To confirm the expression pattern of *CaTIFY4b* and its target genes in developing seed and leaf samples, we performed quantitative RT‐PCR (qRT‐PCR) analysis of ICC 1882, ICC 4958 and a homozygous line (ICCX‐130026‐P17‐P21‐P6), referred to as CaTIFY4b‐NIL, which contains the *CaTIFY4b*‐ICC 4958 allele (Figure 6c–h and Figure S20c–f). The expression of *CaTIFY4b* was significantly up‐regulated in CaTIFY4b‐NIL seeds compared to ICC 1882 seeds at the embryogenesis and pod‐filling stages (Figure 6c). Notably, the expression of *CaGIF1* and *CaCYCD3;1* genes was considerably up‐regulated in the seeds of CaTIFY4b‐NIL plants relative to ICC 1882 plants at the embryogenesis (WW and WS) and pod‐filling (WS) stages (Figure 6d,e). By contrast, the expression levels of *CaTIFY4b* did not reveal major differences in the leaves of CaTIFY4b‐NIL compared with those of ICC 1882, except for a significant decrease at the reproductive stage under water deficit (Figure 6f). The *CaGIF1* gene expression showed a substantial increase in CaTIFY4b‐NIL leaves compared to ICC 1882 leaves under water deficit, at the pod‐filling stage (Figure 6g). Taken together, these results support a mechanism in which *CaTIFY4b* regulates the expression of *GIF1* and related cell cycle genes under water deficit to modulate the growth of developing organs.

## Discussion

Drought stress limits agricultural productivity in the majority of crops. In the case of chickpea, this causes up to 60% yield reduction (Hajjarpoor *et al*., 2018). Therefore, determining the genetic components underlying the production advantage under the most frequent drought scenarios in the major production regions (Hajjarpoor *et al*., 2018) is crucial for improving the yield potential of chickpea. We previously reported the identification of the ‘*QTL‐hotspot*’ region harbouring major effect QTLs for 12 drought adaptation component traits in chickpea (Varshney *et al*., 2014). In this study, high‐resolution mapping delimited the ‘*QTL‐hotspot*’ region to ~113.04‐kb segment containing 13 putative candidates. However, limited genetic recombination and low genetic diversity associated with bi‐parental populations’ hindered pinpointing candidate gene(s) for target traits. Comprehensive analysis of the chickpea composite collection revealed natural variation in the sequence of three genes (*CaTSJT1*, *CaARD1* and *CaTIFY4b*) within the ‘*QTL‐hotspot*’ region that correlated with seed weight. Subcellular localization and transgenic expression in the model legume *M*. *truncatula* revealed *CaTIFY4b* as the most promising candidate, which may control organ growth and consequently the production advantage under water deficit in chickpea. Downstream targets of *CaTIFY4b* homologs have been identified in model plant species (Ge *et al*., 2016; White, 2006) and transcriptional profiling indicated that homologs of their target genes in chickpea are differentially regulated in the seeds and leaves of *CaTIFY4b*‐NIL relative to ICC 1882 under water deficit. Together with physiological screening using homozygous recombinant lines, this result provides a mechanistic understanding of the processes constitutively controlling seed weight and organ size and its potential effect on production under a particular drought scenario in chickpea.

We report that the natural variation in *CaTIFY4b* contributes to seed weight, organ size and has positive consequences on agronomic production under drought in chickpea. The majority of group II members of TIFY family proteins are characterized by the TIFY domain that mediates the formation of homo‐ and heteromeric interactions with other transcription factors (Chung and Howe, 2009; Ge *et al*., 2016). A previous study showed that mutations in the TIFY motif of the Jasmonate ZIM‐domain 10.4 (JAZ10.4) protein blocked JAZ10.4‐JAZ interactions (Chung and Howe, 2009). Therefore, we predict that the ICC 4958 allele of *CaTIFY4b*, which contains a non‐synonymous point mutation (I149S) in the TIFY motif, might interfere with such protein–protein interactions and regulate the expression of the key downstream gene(s) influencing seed and organ size.

In this study, an increase in plant vigour (canopy growth) and TE during early crop development enhanced pre‐anthesis water use and, at least in part, contributed to an increase in seed yield under a specific drought scenario. The most plausible explanation for the present data would be that an early vigour (constitutively enhanced root and shoot growth) was linked to the expression of the *CaTIFY4b*‐ICC 4958 allele, which may have enhanced the carbon supply‐demand balance. This increased pool of assimilates was then translocated to the seeds more effectively, with the moisture available during the crop cycle, resulting in higher yields under water deficit. The current findings are consistent with previous studies, which suggest that introgression of the ‘*QTL‐hotspot*’ substantially improves root growth and architecture of the resulting introgression lines compared to parental lines (Bharadwaj *et al*., 2021; Varshney *et al*., 2013b). It is also in line with our results following ectopic expression of *CaTIFY4b*‐ICC 4958 allele in *M*. *truncatula* hairy roots.

A prolonged period of cell division, leading to increased cell number in the seed coat, cotyledon or endosperm is usually associated with higher seed weight/size in chickpea (Garg *et al*., 2017). Indeed, the GO analysis revealed enrichment of terms such as anisotropic cell growth and regulation of cellulose biosynthesis, in ICC 4958 at the pod‐filling stage. In *Arabidopsis*, *GIF1* and *CYCD3* genes positively regulate primary cell proliferation (Dewitte *et al*., 2007; Liu *et al*., 2020). Our data show that, in CaTIFY4b‐NIL, the increased lateral organ size may be due to prolonged primary cell proliferation, which is consistent with the significant up‐regulation of *CaGIF1* and *CaCYCD3;1* genes in developing seeds and *CaGIF1* gene in leaves. Our results suggest that *CaTIFY4b* may control organ size by modulating *GIF1* gene expression in developing organs, a mechanism similar to that of its ortholog BS1 in *M. truncatula* (Ge *et al*., 2016). The expression of introgressed genes in CaTIFY4b‐NIL is an outcome of the interaction between (i) *trans*‐regulatory factors from ICC 1882 and (ii) *cis*‐regulatory elements in the introgressed ‘*QTL‐hotspot*’ region. The introgression of ‘*QTL‐hotspot*’, in turn, may influence background gene expression. Therefore, transgressive expression of *CaTIFY4b* gene (and some of its downstream target genes) in CaTIFY4b‐NIL can be attributed to the presence of epistatic interactions between the alleles (Rieseberg *et al*., 1999; Sabaghpour *et al*., 2003).

Taken together, fine mapping of the ‘*QTL‐hotspot*’ region and functional characterization of prioritized genes led to the identification of *CaTIFY4b* as a major contributor to the constitutive control of seed weight and organ size, with positive consequences on production under particular drought scenarios. A comprehensive understanding of the physiological and molecular processes underlying *CaTIFY4b* may serve as a valuable resource for uncovering the yield potential and drought adaptation mechanisms in chickpea. As an important regulator of seed weight and organ size, the *CaTIFY4b* gene (and its homologs in other crops) can be a direct target for genetic manipulation and selection to enhance seed yield under drought stress.

## Materials and methods

### Plant material

A chickpea landrace, ICC 1882 and an advanced cultivar, ICC 4958, were used as low and high seed weight genotypes, respectively. ICC 4958 is a drought adapted line that carries the ‘*QTL‐hotspot*’ region, and ICC 1882 is a drought susceptible landrace from India. To characterize the ‘*QTL‐hotspot*’ region, we developed 50 BC_6_F_4_ homozygous recombinant lines segregating for the ‘*QTL‐hotspot*’ by repeated backcrossing with ICC 1882 and marker‐assisted selection to eliminate non‐target DNA regions.

To identify natural variation within genes from the ‘*QTL‐hotspot*’ region, a total of 1712 desi accessions from chickpea composite collection (Upadhyaya *et al*., 2006; Varshney *et al*., 2021d) were targeted. Based on the phenotyping data for 100‐seed weight, we selected 20 accessions from each extremity containing the lowest and highest values. A total of 40 chickpea accessions were used to prioritize candidate genes from the delimited ‘*QTL‐hotspot*’ region.

### Development of homozygous recombinant lines for fine mapping of ‘*QTL‐hotspot*’

We performed crossing between ICC 4958 and ICC 1882 and carried out repeated backcrossing with ICC 1882 genotype as the recipient parent to transfer the genomic region containing ‘*QTL‐hotspot*’ into ICC 1882 genotype. We screened the resulting introgression lines with six simple‐sequence repeat (SSR) markers flanking the ‘*QTL‐hotspot*’ region. We obtained a near isogenic line homozygous for the ICC 4958 allele of the ‘*QTL‐hotspot*’ (ICCX‐110125‐P18; BC_5_F_1_ generation) (Table S1). The ICCX‐110125‐P18 line was used as the donor for backcrossing with ICC 1882 plants to develop BC_6_F_1_ plants (*n* = 73), which were screened with two SSR markers (TAA170 and NCPGR21) flanking the ‘*QTL‐hotspot*’ region. Further, selected BC_6_F_1_ plants were self‐pollinated to generate 1911 BC_6_F_2_ plants for high‐resolution mapping of the ‘*QTL‐hotspot*’. We selected BC_6_F_2_ plants in which recombination occurred within the ‘*QTL‐hotspot*’ region. These plants were self‐pollinated and the progeny (BC_6_F_4_ and BC_6_F_5_) was utilized for fine mapping and characterization of the ‘*QTL‐hotspot*’ region.

### Development and utilization of KASP markers

In order to identify recombination events within the ‘*QTL‐hotspot*’ region, selective SNP markers within ‘*QTL‐hotspot_a*’ and ‘*QTL‐hotspot_b*’ sub‐regions were converted into KASP markers. Marker nomenclature was given as chickpea KASP assay markers (CKAMs). In the first instance, a set of six KASP markers (CKAM2210, CKAM2177, CKAM2178, CKAM2179, CKAM2181 and CKAM2182) were developed and used for screening 1911 BC_6_F_2_ plants (Table S2). Here, CKAM2210 and CKAM2178 were used as flanking markers for ‘*QTL‐hotspot_a*’, whereas CKAM2179 and CKAM2182 were used as flanking markers for ‘*QTL‐hotspot_b*’. Screening of 1911 BC_6_F_2_ plants with six KASP markers identified 42 heterozygous recombinants, of which 15 were identified for ‘*QTL‐hotspot_a*’ whereas 11 recombinants were identified for ‘*QTL‐hotspot_b*’. Moreover, 16 plants were found to possess recombination events within both sub‐regions. The selected F_2_ recombinants were self‐pollinated to develop BC_6_F_3_ plants. To identify more recombination events, a next set of 18 KASP makers (CKAM2211, CKAM2213, CKAM2214, CKAM2215, CKAM2216, CKAM2217, CKAM2218, CKAM2219, CKAM2220, CKAM2221, CKAM2222, CKAM2223, CKAM2224, CKAM2225, CKAM2226, CKAM2227, CKAM2228 and CKAM2229) was developed between the flanking markers CKAM2210 and CKAM2182, to screen 284 BC_6_F_3_ plants (Table S2). This identified 50 homozygous recombinant BC_6_F_3_ lines grouped into 10 families (BC_6_F_3__1 to BC_6_F_3__10), where each family represented one BC_6_F_2_ individual from which the BC_6_F_3_ plants were developed.

### Evaluation of variation index

Genomic sequences of 13 annotated genes from the delimited ‘*QTL‐hotspot*’ region were extracted and used for the analysis. The presence of allelic variation between the germplasm lines belonging to extreme bulks was determined after aligning their sequence data to the reference genome assembly (CaGAv1.0) (Varshney *et al*., 2013a). The variation index value was used to prioritize candidate genes regulating the differences in 100‐seed weight across two extreme bulks. Variation index, a measure of nucleotide diversity at a given genomic position, was counted based on the number of genotypes harbouring SNPs different from the reference sequence (ICC 4958). The genes with a variation index <0.3 in both the bulks were filtered out from the further analysis. If a variation index >0.3 was present in any one of the two bulks, the SNP was considered for further analysis irrespective of the variation index of the other bulk. ∆Variation index was calculated as the difference in the variation index values of the highest bulk and the lowest bulk. A positive ∆Variation index value specifies that the non‐reference allele contributed to the trait, whereas a negative ∆Variation index value indicates that the reference allele contributed to the trait.

### Sequence analysis and gene function prediction

DNA sequences were gathered and aligned using BioEdit software (http://www.mbio.ncsu.edu/bioedit/bioedit.html). Prediction of biological function and the conserved domains of CaTIFY4b protein were performed using InterPro (http://www.ebi.ac.uk/interpro/) and PROSITE (http://www.expasy.org/prosite/) databases.

### Vector construction

Total RNA was extracted from the young leaves of ICC 4958, and the first strand cDNA was synthesized using the SuperScript® III First‐Strand Synthesis System (Invitrogen; Thermo Fisher Scientific, Waltham, Massachusetts, USA). Molecular cloning of prioritized genes was performed using Gateway cloning technology (Curtis and Grossniklaus, 2003), following the manufacturer’s instructions. In brief, the coding sequence of *CaTSJT1*, *CaARD4* and *CaTIFY4b* was amplified from the cDNA of ICC 4958 by polymerase chain reaction (PCR) using primers containing compatible adapters. The PCR product of *CaTSJT1* and *CaARD4* was cloned into Gateway donor vector pDONR 221, whereas that of *CaTIFY4b* was cloned into donor vector pDONR207, by Gateway BP reaction. Plasmids from confirmed positive pDONR clones were cloned into the destination vector (pB7FWG2) using Gateway LR reaction. The cloned plasmids were then transformed into *E. coli* strain DH5α to propagate the plasmid DNA. The colonies were screened using colony PCR and confirmed by sequencing. The *pMtBG2:GUS* construct, carrying DsRed as a marker for transformation driven by ubiquitin (UBIQ) promoter, was used as a control vector. The *pMtBG2:GUS* vector was constructed as described previously (Gaudioso‐Pedraza *et al*., 2018).

### Subcellular localization of genes

We cloned the target proteins in a C‐terminal fusion with a GFP to generate expression vectors driven by the CaMV35S promoter and to investigate the subcellular localization. The *p35S:CaTSJT1‐GFP*, *p35S:CaARD1‐GFP* and *p35S:CaTIFY4b‐GFP* constructs were developed and transformed into ElectroMAX *A. tumefaciens* strain LBA4404 (Invitrogen; Thermo Fisher Scientific, Waltham, Massachusetts, USA). These constructs were then introduced into the fully expanded and intact leaves of *N*. *benthamiana* by agroinfiltration. In brief, the leaves were infected with an inoculum of *A. tumefaciens* (OD_600nm_ 0.6–0.8) suspended in 10 mm MgCl_2_ and 150 μm acetosyringone. After 48 h of incubation at 25 °C, the abaxial side of the transformed leaves was observed under a confocal laser scanning microscope for the presence of GFP and DsRed fluorescence.

### *M. truncatula* transformation and selection of transgenic roots

*Medicago truncatula* (A17) wild‐type seeds were used in this study. *M. truncatula* seeds were scarified using sandpaper to penetrate the seed coat gently. The seeds were surface sterilized for 2 min using diluted bleach water (5% sodium hypochlorite), washed and then incubated for 3 h in sterile water. Sterilized seeds were germinated on inverted Campbell agar plates for 3–4 days at 4 °C and overnight at 25 °C before being used for *A. rhizogenes* transformation. The *A. rhizogenes* strain AR1193 (Stougaard *et al*., 1987) was used for *A. rhizogenes*‐mediated transformation of the *p35S:CaTSJT1‐GFP*, *p35S:CaARD1‐GFP* and *p35S:CaTIFY4b‐GFP* constructs. Further, *A. rhizogenes* strain ARquA1 (Quandt, 1993) was used for *A. rhizogenes*‐mediated transformation of *pMtBG2:GUS* construct. These strains were propagated on selective tryptone yeast media complemented with 6 mmol/L calcium chloride and selective antibiotics. The *A. rhizogenes* AR1193 strain containing binary vector with antibiotic resistance was grown in the presence of 100 µg/mL streptomycin and 100 µg/mL spectinomycin (binary vector resistance antibiotic), whereas the ARquA1 strain was grown in the presence of 100 µg/mL streptomycin and 25 µg/mL chloramphenicol.

*Agrobacterium rhizogenes*‐mediated transformation of *M. truncatula* roots was performed as previously described (Gaudioso‐Pedraza *et al*., 2018), with minor changes. In brief, germinated seedlings with a radicle length of approximately 8–10 mm were sectioned at 3 mm from the root tip and the cut end was inoculated by dipping in a suspension of *A. rhizogenes* (OD_600nm_ 0.6–0.8). The seedlings were then placed on FP agar plates (12 cm × 12 cm) supplemented with ammonium nitrate (NH_4_NO_3_; 0.25 mm), maintaining a density of 10 seedlings plate^−1^. Plates were placed vertically in plastic boxes under controlled environments with 16 h light/8 h dark photoperiods at 20 °C for 3 weeks. *M. truncatula* hairy roots were selected using an Olympus BX61 fluorescence microscope for the expression of GFP and DsRed fluorescent proteins encoded in the T‐DNA of the target gene and control transformation binary vectors, respectively. Individual GFP^+^ and DsRed^+^ composite plants were viewed under a confocal microscope for ectopic expression and were used for drought experiments.

### Drought experiments with transgenic *M. truncatula*

Transformed *M. truncatula* plants were grown in plastic cups (23 cm diameter and 26 cm height) filled with a mixture of Terragreen:sand:perlite (3:1:1, w/w) as substrate, under controlled conditions (16‐h day/8‐h night; 20 °C/16 °C day/night temperature). Plants were watered with half strength Fahraeus nutrient solution containing 0.125 mm ammonium nitrate for the first week to improve plant performance during the initial growth stage. After about 7 days, the plants were randomly separated into two sets: WW and WS. The WW set of plants was daily supplied with water, whereas the WS set was subjected to progressive drought by withholding water for 8 days. All the plants were subsequently watered, and the survival rate of the transgenic plants was calculated 1 week after rehydration. Plants with green and viable leaves and stems were considered as survivors. After the drought treatment, the roots were gently washed with water and the plants were phenotyped for root depth, root fresh weight, shoot fresh weight and total fresh weight. The root depth of the seedlings was measured using ImageJ software (https://imagej.nih.gov/ij/).

### Microscopy methods

*Medicago truncatula* roots were excised about 2 cm from the root tip and mounted in propidium iodide solution (1:100 dilution). Micrographs were taken at the root tip and the differentiation zone. Confocal imaging analysis was conducted using a Zeiss LSM700 inverted and LSM800 upright microscope using a 488 nm excitation laser for GFP or a 550 nm for propidium iodide and DsRed. Emission was received using the filters BP 505‐530 for GFP and BP 600‐630 filter for propidium iodide. The images captured resembled individual stacks of z‐optical sections. Images were acquired, projected and processed using imaging software ZEN version 2.6 (Zeiss, Oberkochen, Germany).

### Evaluation of agronomic traits under rainfed field conditions

The homozygous recombinant lines and parental lines (ICC 1882 and ICC 4958) were grown under rainfed field conditions at ICRISAT, India, in the post‐rainy season of 2017–18 and 2018–19. The experiment was laid out in an alpha lattice design with three replications of each genotype. Each plot comprised a single 4 m row with a spacing of 60 cm between rows and 10 cm between plants within the same row. Sixty seeds were sown in each 4 m row and later maintained to 40 plants per row after seedling establishment. Optimal irrigation was provided immediately after sowing to ensure a uniform emergence of seedlings. The plants received no irrigation further and were subsequently grown under rainfed conditions. During the 2017–18 crop season, the average day/night temperature recorded was 30.7/13.2 °C with a relative humidity of 31.7/80.8%. The average day/night temperature and relative humidity during the 2018–19 crop season were 30.1/15.3 °C and 45.0/95.2%, respectively.

This experiment mainly focused on the evaluation of agronomic traits (Table S5). Five healthy plants were selected randomly from each row at physiological maturity to measure seed yield and its components. The scoring of plant vigour was performed at the vegetative stage visually on a scale of 1–5, where late vigour parent (ICC 1882) and early vigour parent (ICC 4958) were assigned a score of 2 and 5, respectively (Sivasakthi *et al*., 2018). Residual yield not explained by flowering and harvest index (HI) was calculated as the difference between the observed and predicted yields, as described previously (Tharanya *et al*., 2018). We defined harvest index as the ratio between seed yield per plant and shoot biomass per plant.

### Phenotyping for water uptake traits at the rain‐out shelter

A total of 21 genotypes, including 19 homozygous lines (two lines from each of the 10 families, except BC_6_F_3__4) and two parental lines, were grown in a rain‐out shelter at ICRISAT, India (http://gems.icrisat.org/lysimetric‐facility/) in the post‐rainy season of 2017–18 and 2018–19. A randomized block design with treatment as the main factor and genotype as the sub‐factor consisting of five replications per block was used. The plants were grown in lysimeters, consisting of polyvinyl chloride (PVC) cylinders (120 cm height and 20 cm diameter), according to the method described previously (Zaman‐Allah *et al*., 2011). In brief, the cylinders were filled with about 42 kg of vertisol, which was fertilized with di‐ammonium phosphate and single super phosphate at the rate of 100 and 300 mg/kg soil, respectively. Four seeds per genotype were sown and two plants per cylinder were maintained at 15 DAS. All the plants were grown under normal conditions until the imposition of drought stress. One block was assigned to WW treatment and the other block to WS treatment. Prior to the imposition of WS treatment, all the cylinders were completely saturated with water and allowed to drain overnight. The next morning, the top layer of the soil was covered with a round plastic sheet, above which a 2 cm thick layer of polyethylene granules was laid. The WS treatment was imposed by withholding water from the vegetative stage (29 DAS in 2017–18 and 27 DAS in 2018–19) until harvest. The WW set of plants was irrigated weekly to retain the soil water levels above 80% of the field capacity. Plant water uptake on a weekly basis was calculated based on the losses in the weight of each cylinder. When the net transpiration ratio (NTR) of the WS plants fell below 0.10, that is, when the transpiration value of WS plants was <10% of that of WW plants, the WS set of plants was harvested. The WW set of plants was harvested at physiological maturity. After harvest, the plants were dried in the oven for 3 days at 70 °C and yield, yield components and biomass traits were recorded per cylinder basis. The pre‐anthesis water use was the sum of the daily water use values from the imposition of WS treatment until flowering. Post‐anthesis water use was the sum of the daily water use values from the end of flowering until maturity. Transpiration efficiency was estimated as the ratio of total shoot dry weight produced divided by total water extracted.

### Evaluation of root system architecture and water uptake patterns under glasshouse environment

We quantified the root system architecture traits in chickpea plants at 35 DAS, to obtain maximum variation between the genotypes (Kashiwagi *et al*., 2006). A total of 20 homozygous lines (two lines from each of the 10 families) and two parental lines were grown in PVC cylinders (120 cm height and 16 cm diameter) during the post‐rainy season of 2019–20. The experiment was conducted using a complete randomized block design, with five replications of each genotype. One plant per cylinder was maintained until harvest. At 35 DAS, plants were cut at the soil surface. The entire root system was sliced into sections of 30 cm to measure the root length, root length density, root surface area and root volume using the image analysis software (WinRhizo, Regent Instruments Inc., Montreal, Quebec, Canada), as described earlier (Zaman‐Allah *et al*., 2011). We defined root length density as the total root length divided by the volume of the cylinder.

To evaluate root growth and architecture traits and understand the relationship between the capacity of the root to extract soil water and drought adaptation, we conducted experiments in the glasshouse facility at ICRISAT, India, in the post‐rainy season of 2017–18 and 2018–19. Near‐optimal conditions (day/night temperature 32/25 °C and relative humidity varying between 40% and 80% during the day) were maintained in the glasshouse during the experiment. The plants were grown in lysimeters, comprising PVC cylinders (120 cm height and 20 cm diameter) and the soil type, fertilizer application and protocol used for the imposition of drought stress were the same as described above for rain‐out shelter lysimeters. A complete randomized block design, comprising treatments as the main factor (one block in 2017–18 and two blocks in 2018–19) and genotype as the sub‐factor that was replicated three times within each block was used for the experiment. Two plants were maintained per cylinder after seedling establishment until harvest. The WS treatment was imposed by the termination of watering from 27 DAS (2017–18 and 2018–19). The WS set of plants was harvested when the NTR of stressed plants fell below 0.10, as described above, and the yield, yield components and shoot biomass traits were measured. Further, the soil from the cylinders was washed to obtain the entire root profile. Root phenotyping and data analysis were performed as described above.

### High‐throughput phenotyping of canopy development traits

An imaging platform (LeasyScan), based on a 3D imaging technique, was used to assess the canopy development traits during the vegetative stage of the crop (Vadez *et al*., 2015). Fifty homozygous recombinant lines and two parental lines were grown under fully irrigated conditions in two experiments conducted during December–January of 2017–18 and 2018–19, at ICRISAT, India (http://gems.icrisat.org/leasyscan/). The Phenospex plant eye scanners imaged a preset area of 65 × 40 cm regarded as a ‘sector’. A final count of eight plants per sector (~0.25 m^2^ area) was maintained, equivalent to the sowing density of 32 plants/m^2^, which is comparable to the sowing density of chickpea in the fields. The experimental design was a randomized block design. Di‐ammonium phosphate was applied at a rate of 300 mg/kg of soil. The specific leaf area (one‐sided area of the leaf) was estimated by dividing the 3D‐leaf area with shoot dry weight, and specific leaf weight was calculated as the reciprocal of specific leaf area (1/specific leaf area). The canopy structure was calculated as described earlier (Tharanya *et al*., 2018). The growth rates for 3D‐leaf area and projected leaf area were calculated during the exponential phase of the plant growth, as described previously (Sivasakthi *et al*., 2018). The average day/night temperature recorded during the crop growth was 31/5.2 °C with a relative humidity of 26/99%.

### Statistical analysis of the phenotypes

Analysis of variance was computed to test the significance of genotype (G), environment (E) and genotype × environment (G × E) interactions using the statistical software GenStat v15.0 (VSN International, Hemel UK). The differences between genotype means were analysed with Tukey’s test (at 0.05 significance level) or two‐sided *t*‐test, using GenStat (v15.0). Dunnett’s test was performed using RBioplot package of R (R Project for Statistical Computing) (Zhang and Storey, 2016). The residual maximum likelihood method in GenStat (v15.0) was used to obtain an unbiased estimate of the variance components and the best linear unbiased prediction (BLUP) of different parameters within the treatment, considering genotypes as random effects and replications as fixed effects. The BLUP estimates were used for generating PCA biplots using FactoMineR package of R. Repeatability of the phenotype was based on broad‐sense heritability (*h* ^2^), which was estimated as *h* ^2^ = $\sigma_{g}^{2}$ / ($\sigma_{g}^{2}+\sigma_{e}^{2}$), where $\sigma_{g}^{2}$ is the genetic variance, and $\sigma_{e}^{2}$ is the residual error (Tharanya *et al*., 2018).

### RNA *isolation* and RNA sequencing

The parental lines (ICC 4958 and ICC 1882) and a homozygous line (ICCX‐130026‐P17‐P21‐P6) containing the CaTIFY4b‐ICC 4958 allele, regarded as CaTIFY4b‐NIL, were selected. The plants were grown in pots in a glasshouse under near‐optimal conditions (day/night temperature 32/25 °C and relative humidity oscillating between 40%–80% during the day). Three biological replicates were maintained per genotype at each stage and water treatment for tissue collection. Plants were exposed to two water treatments: WW and WS, and WS treatment was imposed at 25 DAS. The WW set of plants was maintained at ~80% field capacity. The WS set of plants was subjected to a progressive drought stress by partially recompensing water loss from transpiration. The leaf samples were harvested at the reproductive stage. The immature seeds were collected about 10 days after the initiation of flowering, which represented the embryo development stage. At the pod‐filling stage, when the NTR of WS plants dropped below 0.20, the seed and leaf samples were harvested. All the samples were flash frozen in liquid nitrogen and stored at −80 °C for RNA isolation.

Total RNA was extracted from the seed and leaf samples using the ‘NucleoSpin® RNA Plant’ kit (Macherey‐Nagel, Dueren, Germany), following the manufacturer’s instructions. The qualitative and quantitative assessment of these RNA samples was conducted using the Qubit RNA assay kit (Thermo Fisher Scientific, Waltham, Massachusetts, USA) and BioAnalyzer 2100 (Agilent Technologies, Santa Clara, California, USA). The high‐quality total RNA was used for cDNA library construction, and RNA‐seq was performed using Illumina HiSeq 2500 platform at Centre of Excellence in Genomics & Systems Biology, ICRISAT (https://cegsb.icrisat.org/). A total of 16 samples representing seed and leaf tissues of ICC 4958 and ICC 1882 collected at two different stages under WW and WS conditions, in two replicates, were used for sequencing. The raw reads were filtered to remove adaptors, low‐quality bases and reads using Trimmomatic v0.39 (Bolger *et al*., 2014). The filtered reads were mapped to the draft sequence of chickpea genome (CaGAv1.0) (Varshney *et al*., 2013a) using HISAT2 (v2.2.1) (Kim *et al*., 2019). The StringTie (v2.1.4) package (Pertea *et al*., 2015) was used to assemble mapped reads from each sample to produce reference‐guided assemblies and subsequently a consensus transcriptome assembly. The DEGs were identified using DESeq2 package (Love *et al*., 2014). A particular gene was considered differentially expressed in a pairwise comparison between ICC 4958 and ICC 1882 samples if it exhibited log_2_ fold change of ≥1 or ≤ –1 and corrected *P*‐value ≤0.05.

### Gene annotations, GO *terms* and pathway enrichment analysis

To determine their putative functions, the identified DEGs were subjected to Blastx similarity searches (*E*‐value < 1e‐05) against NCBI non‐redundant protein databases. GO enrichment analysis for DEGs was performed using the R‐based GOseq package (Young *et al*., 2010). Furthermore, the MapMan bin annotations for the genes were obtained using Mercator4 v2.0 (Schwacke *et al*., 2019). The GO terms and MapMan bins exhibiting a corrected *P*‐value ≤0.05 were considered significantly enriched for a particular set of genes.

### Candidate gene *expression* analysis

RNA was isolated from the seed and leaf samples of ICC 4958, ICC 1882 and CaTIFY4b‐NIL, collected at the reproductive and pod‐filling stages, under WW and WS conditions. The first‐strand cDNA synthesis was performed using the SuperScript® III First‐Strand Synthesis System (Invitrogen; Thermo Fisher Scientific, Waltham, Massachusetts, USA), following the manufacturer’s instructions. Quantitative real‐time PCR (qRT‐PCR) was performed on Applied Biosystems 7500 Real‐Time PCR System using SYBR Green (Applied Biosystems, Waltham, Massachusetts, USA). Gene‐specific primers used for qRT‐PCR were designed using ‘Primer 3’ software (Rozen and Skaletsky, 2000), and the primer sequences are listed in Table S2. The glyceraldehyde 3‐phosphate dehydrogenase (*GAPDH*) gene was used as an endogenous control. The data obtained from different cDNA samples were compared using the mean *C*_t_ values of three biological replicates, which were normalized to the mean *C*_t_ values of the endogenous control. The relative gene expression was measured using the $2^{‐\Delta \Delta C_{t}}$ method, and the significance value was calculated using Student’s *t‐*test.

## Conflict of interest

The authors declare no competing interests.

## Author contributions

R.K.V. conceived and designed the experiments. Y.B.‐A. contributed to the design of constructs and experiments involving *M*. *truncatula*. R.B. performed *M. truncatula* and *N*. *benthamiana* transformation and transgenic analysis, carried out phenotyping of homozygous recombinant lines at different experimental platforms, identified the *CaTIFY4b* gene and analysed gene expression levels. V.G. and A.W.K. analysed the RNA‐seq data and sequencing data of chickpea germplasm accessions, respectively. Together with R.B., L.G. made all GFP‐fusion constructs and carried out *M. truncatula* and *N. benthamiana* transformation, intracellular localization experiments and phenotyped transgenic *Medicago* roots in soil and plates. D.J. and S.S. carried out the development of homozygous recombinant lines. D.J., S.M.K., R.B. and M.R. contributed to the development and utilization of KASP markers. R.B., M.R., V.G., A.W.K., L.G., D.J., J.K., H.K., S.K., S.M.K., Y.B.‐A. and R.K.V analysed the data. M.R., A.C., J.K., H.K., S.S., P.M.G., S.R.S., Y.B.‐A. and R.K.V contributed to the reagents, materials, generation of various kind of data sets and analysis tools. R.B. wrote the manuscript along with M.R., Y.B.‐A. and R.K.V. All authors read and approved the manuscript.

## Supporting information


**Figure S1** Genotype classes of BC_6_F_2_ recombinant lines segregating for ‘*QTL‐hotspot_a*’ and ‘*QTL‐hotspot_b*’ sub‐regions.
**Figure S2** Representative snapshots depicting SNP genotyping using KASP markers.
**Figure S3** Characterization of seed weight in ICC 1882‐ and ICC 4958‐homozygous lines of ‘*QTL‐hotspot*’
**Figure S4** Comparison of root growth and architecture traits measured at 35 days after sowing for plants grown in lysimeters.
**Figure S5** Comparison of predicted amino acid sequences between *CaTSJT1* and its homologs in other legume plants.
**Figure S6** Comparison of deduced amino acid sequences between *CaTIFY4b* and its homologs in other legume plants.
**Figure S7** Predicted protein structure of CaTIFY4b and amino acid sequence alignment of the TIFY domain from extreme chickpea germplasm accessions.
**Figure S8** Evaluation of drought adaptation component traits in transgenic *Medicago truncatula*.
**Figure S9** Effect of ‘*QTL‐hotspot*’ on water use‐related traits evaluated at the rain‐out shelter environments.
**Figure S10** Phenotypic characterization of root growth and architecture traits evaluated at pod‐filling stage of crop growth.
**Figure S11** Effect of ‘*QTL‐hotspot*’ on canopy development traits phenotyped at LeasyScan platform and under field conditions.
**Figure S12** Time course analysis and variation in the growth rate of 3D‐leaf area and projected leaf area.
**Figure S13** Principal component analysis and correlation analysis for traits measured under field conditions.
**Figure S14** Principal component analysis for traits measured at rain‐out shelter and field conditions.
**Figure S15** Time course analysis of water uptake profile and relationship between water uptake in pre‐anthesis and post‐anthesis stages.
**Figure S16** Principal component analysis for phenotypic traits evaluated at LeasyScan and rain‐out shelter.
**Figure S17** Principal component analysis for phenotypic traits evaluated under glasshouse environments.
**Figure S18** Principal component analysis and correlation analysis between seed and leaf transcriptomes of parental genotypes at different development stages and water treatments.
**Figure S19** *In silico* analysis of *CaTIFY4b* gene expression.
**Figure S20** Transcriptome and quantitative RT‐PCR analysis of CaTIFY4b downstream target genes in seed and leaf samples.

**Table S1** Background recovery of ICCX‐110125‐P18 line using genome‐wide SSR markers.

**Table S2** Primers used for fine mapping, gene cloning, subcellular localization and gene expression analysis.

**Table S3** Genotypes of 24 KASP markers on the ‘*QTL‐hotspot*’ region in the BC_6_F_3_ lines and 100‐seed weight of the BC_6_F_4_ progeny.

**Table S4** List of annotated genes in delimited ‘*QTL‐hotspot*’ region based on the reference sequence of CDC Frontier.

**Table S5** Summary of phenotypic traits measured at different platforms (field, rain‐out shelter, glasshouse and LeasyScan).

**Table S6** Summary of common and unique traits evaluated at field, rain‐out shelter, glasshouse and LeasyScan platforms.

**Table S7** Variation in traits evaluated at different experimental platforms.

**Table S8** Analysis of variance for traits evaluated across different environments and/or treatments.

**Table S9** MapMan analysis of the differentially regulated genes in the seed and leaf samples.

## Data Availability

The RNA‐Seq data have been deposited in the NCBI Sequence Read Archive with the BioProject ID: PRJNA770524. The phenotyping data that support the findings of this study have been deposited in figshare (https://figshare.com/) with https://doi.org/10.6084/m9.figshare.16830691. The sequences reported in this article have been deposited in the NCBI GenBank database [accession numbers: OK572370 (*CaTSJT1*), OK572371 (*CaARD1*) and OK572372 (*CaTIFY4b*)]. Further information and requests for DNA constructs used to generate *M. truncatula* transgenics should be directed to and will be fulfilled by Yoselin Benitez‐Alfonso (Y.Benitez-Alfonso@leeds.ac.uk).
