# Supplementary material for: Genetic variation in CaTIFY4b contributes to drought adaptation in chickpea
Source: Plant Biotechnol J. 2022 May 21;20(9):1701–15. doi: 10.1111/pbi.13840 (PMC9398337; doi:10.1111/pbi.13840)
Supplement: Supplementary file 1 — Figure S1 Genotype classes of BC6F2 recombinant lines segregating for ‘QTL‐hotspot_a’ and ‘QTL‐hotspot_b’ sub‐regions. Figure S2 Representative snapshots depicting SNP genotyping using KASP markers. Figure S3 Characterization of seed weight in ICC 1882‐ and ICC 4958‐homozygous lines of ‘QTL‐hotspot’ Figure S4 Comparison of root growth and architecture traits measured at 35 days after sowing for plants grown in lysimeters. Figure S5 Comparison of predicted amino acid sequences between CaTSJT1 and its homologs in other legume plants. Figure S6 Comparison of deduced amino acid sequences between CaTIFY4b and its homologs in other legume plants. Figure S7 Predicted protein structure of CaTIFY4b and amino acid sequence alignment of the TIFY domain from extreme chickpea germplasm accessions. Figure S8 Evaluation of drought adaptation component traits in transgenic Medicago truncatula. Figure S9 Effect of ‘QTL‐hotspot’ on water use‐related traits evaluated at the rain‐out shelter environments. Figure S10 Phenotypic characterization of root growth and architecture traits evaluated at pod‐filling stage of crop growth. Figure S11 Effect of ‘QTL‐hotspot’ on canopy development traits phenotyped at LeasyScan platform and under field conditions. Figure S12 Time course analysis and variation in the growth rate of 3D‐leaf area and projected leaf area. Figure S13 Principal component analysis and correlation analysis for traits measured under field conditions. Figure S14 Principal component analysis for traits measured at rain‐out shelter and field conditions. Figure S15 Time course analysis of water uptake profile and relationship between water uptake in pre‐anthesis and post‐anthesis stages. Figure S16 Principal component analysis for phenotypic traits evaluated at LeasyScan and rain‐out shelter. Figure S17 Principal component analysis for phenotypic traits evaluated under glasshouse environments. Figure S18 Principal component analysis and correlation analysis between seed and leaf [file PBI-20-1701-s001.pdf]

Genetic variation in *CaTIFY4b* contributes to drought adaptation in chickpea

Rutwik Barmukh[1,2], Manish Roorkiwal[1,3,4], Vanika Garg[1], Aamir W. Khan[1], Liam German[5], Deepa Jaganathan[1], Annapurna Chitikineni[1], Jana Kholova[6], Himabindu Kudapa[1], Kaliamoorthy Sivasakthi[6], Srinivasan Samineni[7], Sandip M. Kale[1], Pooran M. Gaur[4,7], Someswar Rao Sagurthi[2], Yoselin Benitez-Alfonso[5], Rajeev K. Varshney[1,4,8,*]

[1]Centre of Excellence in Genomics and Systems Biology, International Crops Research Institute for the Semi-Arid Tropics (ICRISAT), Hyderabad, India

[2]Department of Genetics, Osmania University, Hyderabad, India

[3]Khalifa Center for Genetic Engineering and Biotechnology, United Arab Emirates University, Al-Ain, United Arab Emirates

[4]The UWA Institute of Agriculture, The University of Western Australia, Perth, Australia

[5]Centre for Plant Science, School of Biology, University of Leeds, Leeds, UK

[6]Crop Physiology and Modelling, International Crops Research Institute for the Semi-Arid Tropics (ICRISAT), Hyderabad, India

[7]Crop Breeding, International Crops Research Institute for the Semi-Arid Tropics (ICRISAT), Hyderabad, India

[8]Murdoch's Centre for Crop & Food Innovation, State Agricultural Biotechnology Centre, Food Futures Institute, Murdoch University, Murdoch, Western Australia, Australia

[*]Corresponding Author: r.k.varshney@cgiar.org / rajeev.varshney@murdoch.edu.au

Supplementary Figures

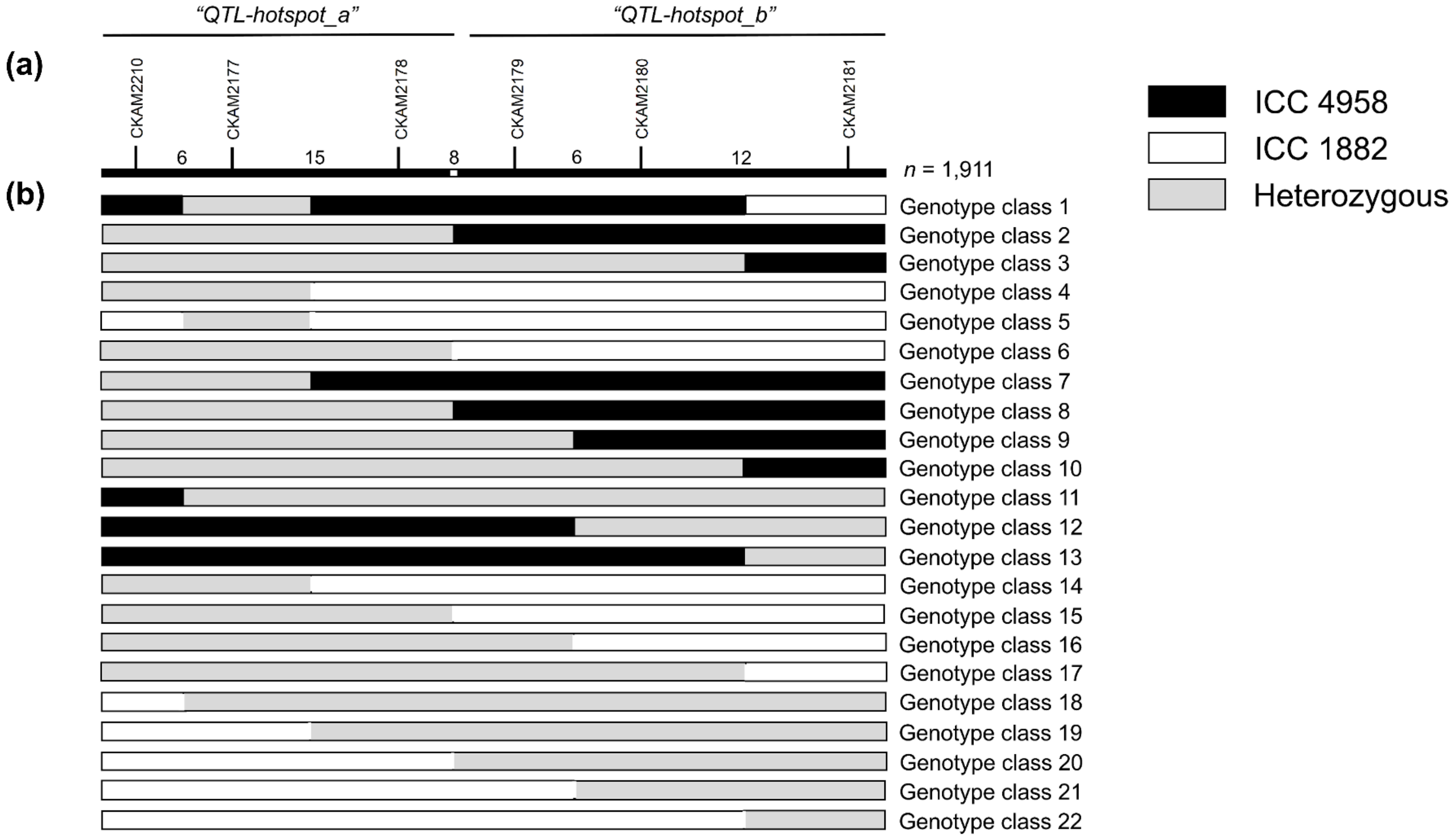

Figure S1. Genotype classes of BC_6F_2 recombinant lines segregating for *"QTL-hotspot_a"* and *"QTL-hotspot_b"* sub-regions.
(a) High-resolution linkage map of *"QTL-hotspot_a"* and *"QTL-hotspot_b"* sub-regions obtained with 1,911 BC_6F_2 plants. The number of recombinants between the adjacent KASP markers is denoted above the linkage map. **(b)** Graphical representation of 22 genotype classes that were observed based on the recombination events that occurred after genotyping of 1,911 BC_6F_2 plants with six KASP markers.

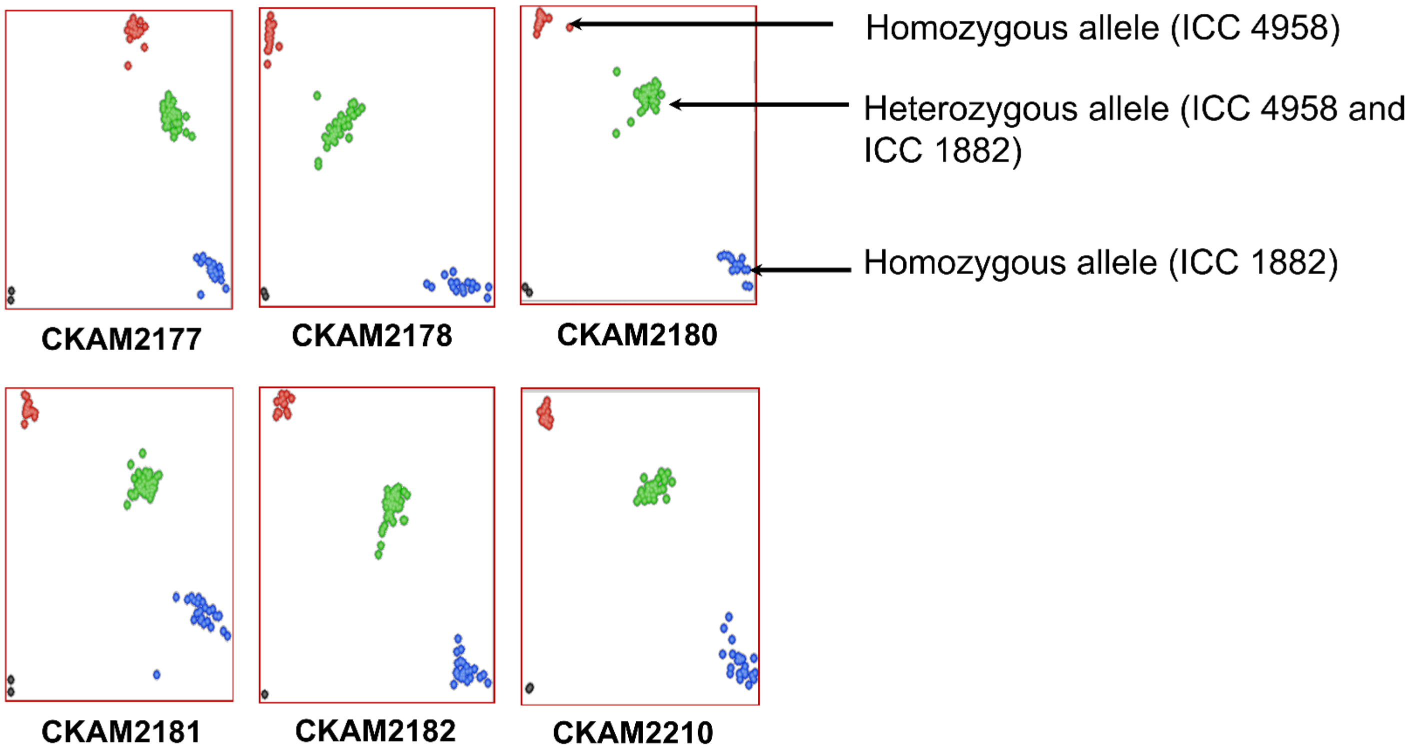

Figure S2. Representative snapshots depicting SNP genotyping using KASP markers.
Marker genotyping data generated for each genotype were used for allele calling. Allelic discrimination (two alleles) for a particular marker in the genotypes examined has been shown on a scatter plot with X and Y axes. Red dots, genotypes containing homozygous alleles of ICC 4958; blue dots, genotypes containing homozygous alleles of ICC 1882; green dots, genotypes containing heterozygous alleles.

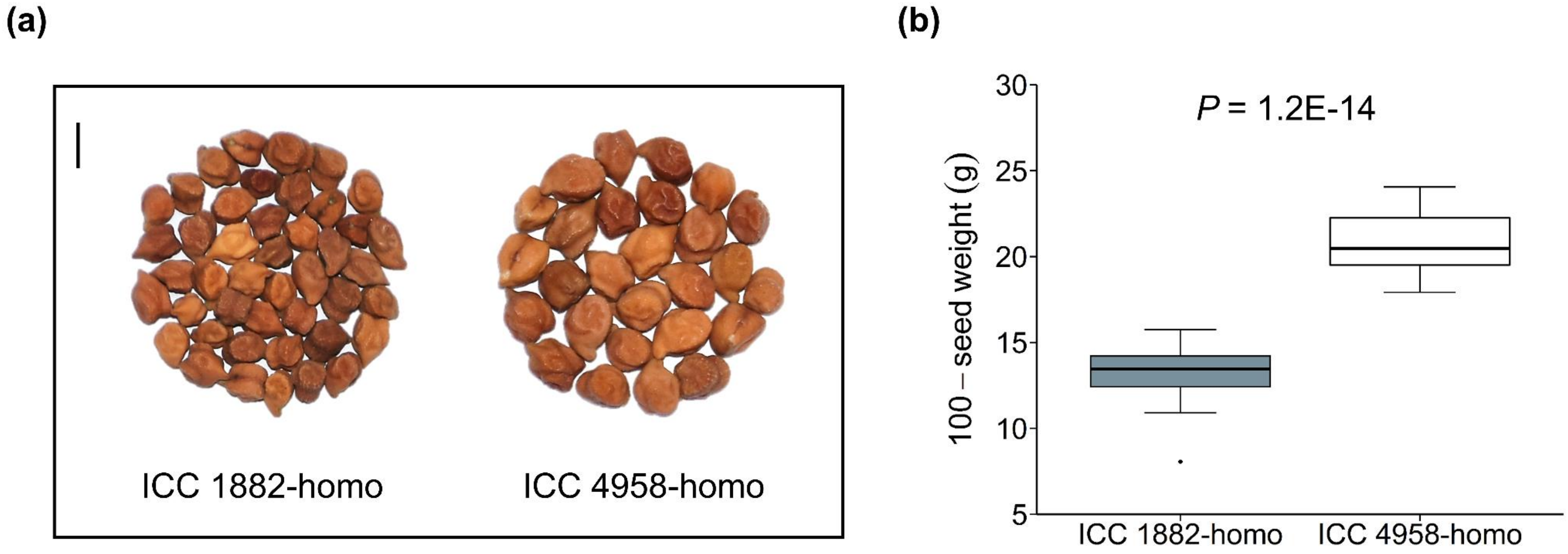

Figure S3. Characterization of seed weight in ICC 1882- and ICC 4958-homozygous lines of *"QTL-hotspot"*.
(a) Images of seeds harvested from ICC 1882-homo and ICC 4958-homo lines. Scale bar, 1 cm. **(b)** Differences in the 100-seed weight among *"QTL-hotspot"* homozygous lines possessing ICC 1882 and ICC 4958 alleles. A total of 34 ICC 1882-homo lines and eight ICC 4958-homo lines were considered for this analysis. For the box plot, boxes represent the 25th-75th percentile, whiskers represent the full data range and the center lines indicate medians. *P* values are based on ANOVA.

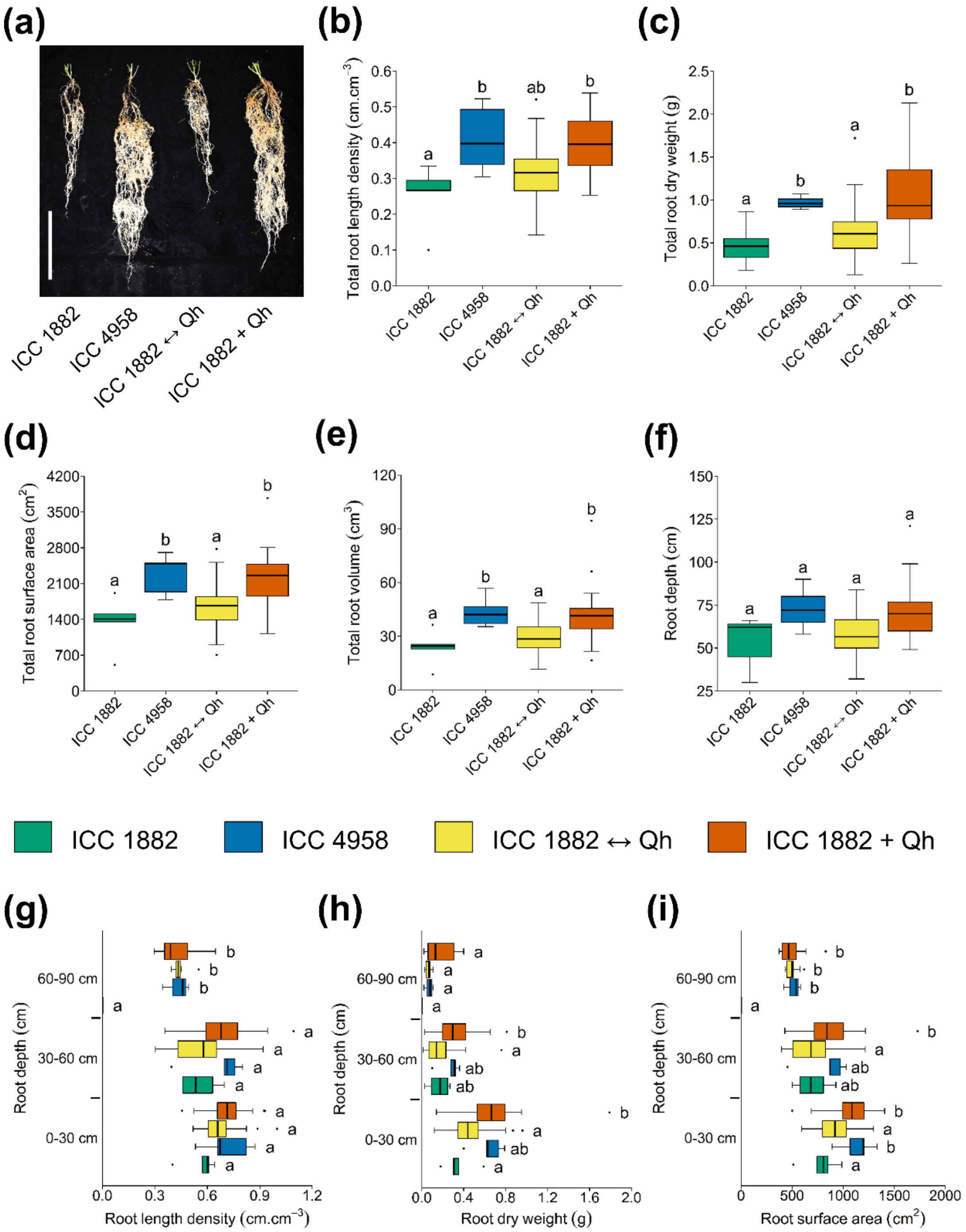

Figure S4. Comparison of root growth and architecture traits measured at 35 days after sowing for plants grown in lysimeters.

(a) Root phenotype of ICC 1882, ICC 4958, ICC 1882 ↔ Qh, and ICC 1882 + Qh plants at 35 days after sowing. Scale bar, 20 cm. **(b)** Total root length density, **(c)** total root dry weight per plant, **(d)** total root surface area, **(e)** total root volume, and **(f)** root depth. **(g-i)** Comparison of **(g)** root length density, **(h)** root dry weight per plant, and **(i)** root surface area, at 30 cm intervals of soil depth for ICC 1882, ICC 4958, ICC 1882 ↔ Qh, and ICC 1882 + Qh plants. The root depth interval for the phenotypic traits evaluated are shown on the y-axis in **(g-i)**. For the box plots, boxes denote the 25th-75th percentile, whiskers denote the full data range and the center lines denote medians. The alphabets above the boxes (a, b) designate statistical significance between the groups of genotypes computed using Tukey's test ($P < 0.05$) in panels **(b-i)**.

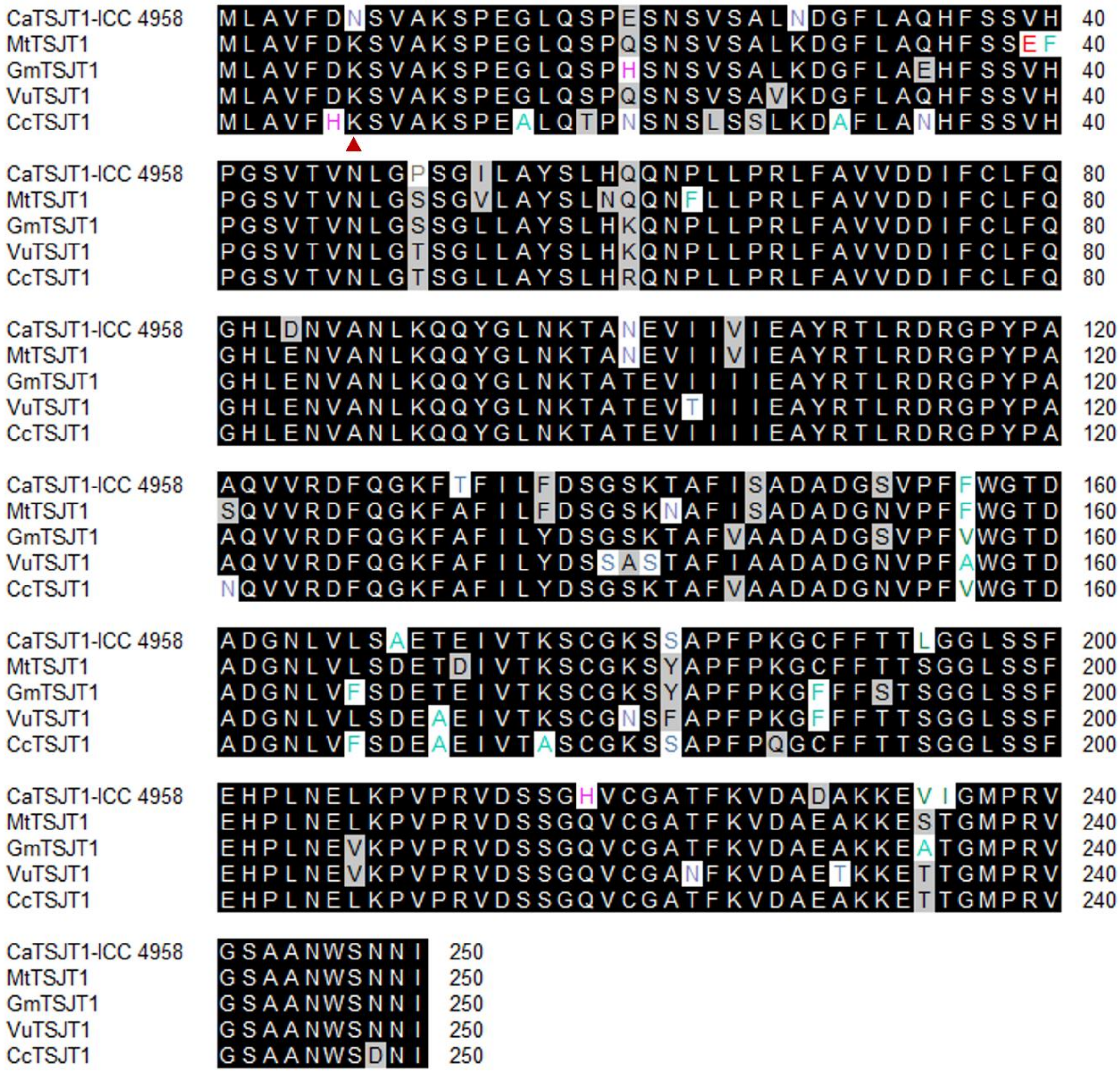

Figure S5. Comparison of predicted amino acid sequences between CaTSJT1 and its homologs in other legume plants.

CaTSJT1-ICC 4958, CaTSJT1 of chickpea cv. ICC 4958. Non-chickpea sequences are MtTSJT1 (*Medicago truncatula;* XP_013469738.1), GmTSJT1 (*Glycine max*; XP_003536502.1), VuTSJT1 (*Vigna unguiculata*; XP_027938177.1), and CcTSJT1 (*Cajanus cajan*; XP_020210176.1). Amino acid residues identical across accessions are represented by black shading. The red arrowhead at the bottom of the sequence indicates the location of a non-synonymous point mutation in ICC 4958.

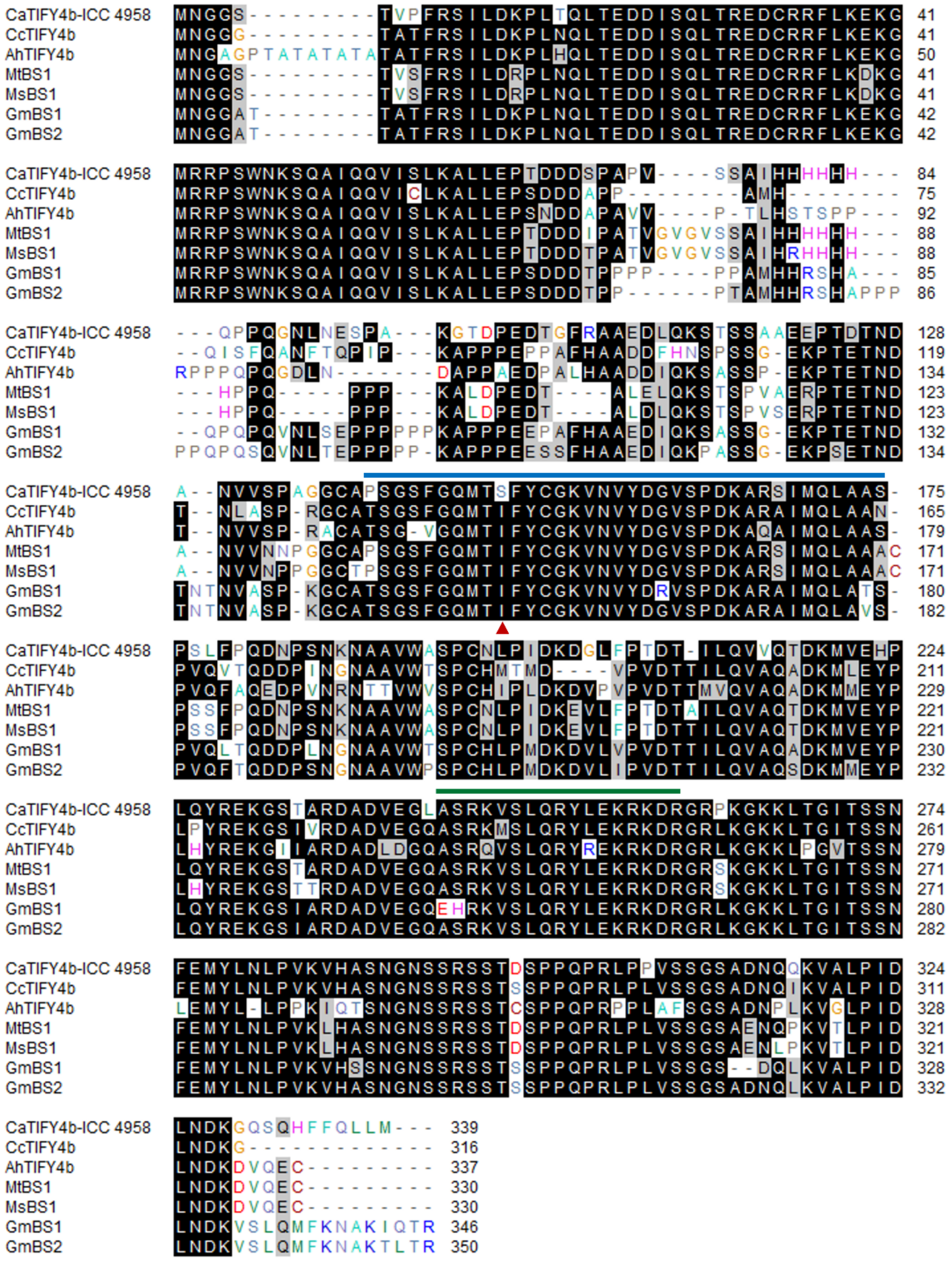

Figure S6. Comparison of deduced amino acid sequences between CaTIFY4b and its homologs in other legume plants.

CaTIFY4b-ICC 4958, CaTIFY4b of chickpea cv. ICC 4958. Non-chickpea sequences are CcTIFY4b (*Cajanus cajan;* KYP74989.1), AhTIFY4b (*Arachis hypogaea*; XP_025620247.1), MtBS1 (*Medicago truncatula*; KM668032), MsBS1 (*Medicago sativa*; KM668033), GmBS1 (*Glycine max*; KM668027), and GmBS2 (*Glycine max*; KM668028). Amino acid residues identical across accessions are represented by black shading. The red arrowhead at the bottom of the sequence indicates the location of a non-synonymous point mutation in ICC 4958. Blue and green bars above the sequence indicate putative TIFY and CCT2 domains, respectively, as predicted by InterPro (http://www.ebi.ac.uk/interpro/).

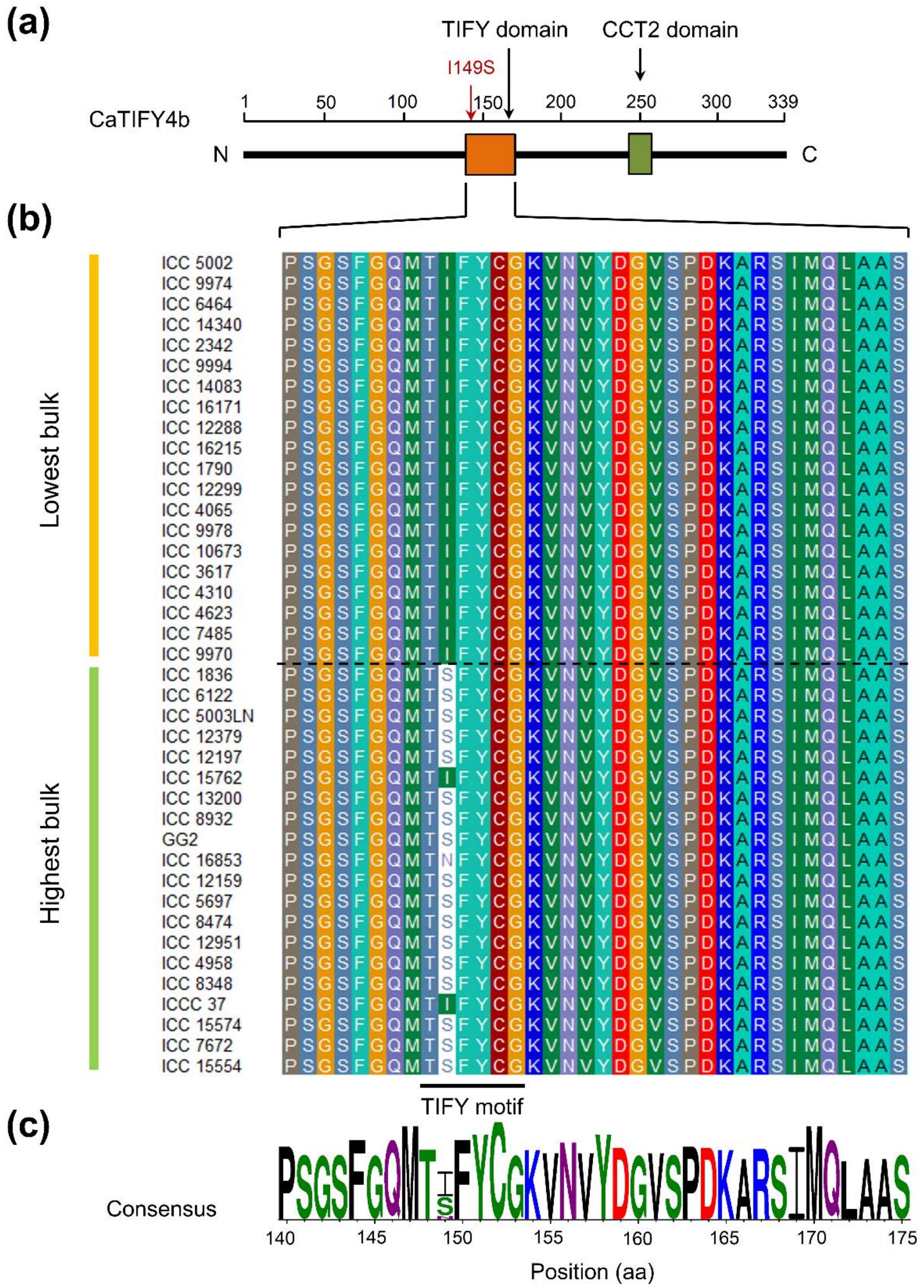

Figure S7. Predicted protein structure of CaTIFY4b and amino acid sequence alignment of the TIFY domain from extreme chickpea germplasm accessions.

(a) Protein structure of CaTIFY4b predicted by InterPro database based on annotation of Ca_04558. Scale on the top indicate amino acid residues. Orange rectangle, TIFY domain; green rectangle, CCT2 domain; red arrow, mutation site; N, N-terminal of the protein; C, C-terminal of the protein. **(b)** Amino acid sequence alignment of the TIFY domain from 40 extreme chickpea accessions belonging to the lowest bulk and highest bulk. Identical residue present at a particular position across all the accessions is highlighted by the same color. **(c)** Representation of sequence logo with conservation patterns at each position in the multiple sequence alignment. Color scheme is based on the chemistry of amino acid residues.

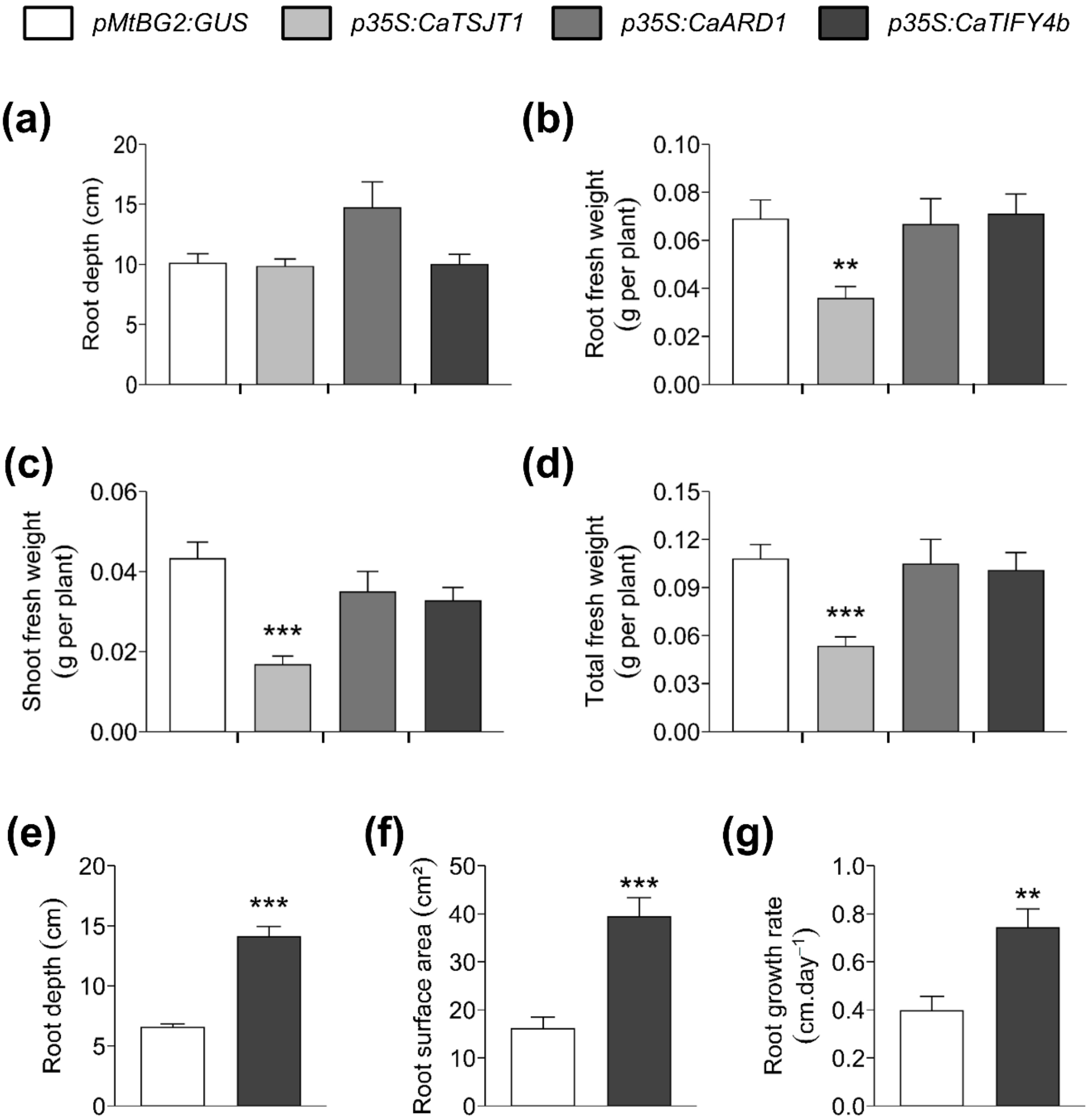

Figure S8. Evaluation of drought adaptation component traits in transgenic *Medicago truncatula*.

(a-d) Statistical comparisons of **(a)** root depth, **(b)** root fresh weight, **(c)** shoot fresh weight, and **(d)** total fresh weight for *pMtBG2:GUS*, *p35S:CaTSJT1*, *p35S:CaARD1*, and *p35S:CaTIFY4b* transformed hairy roots under well-watered conditions. **(e-g)** Statistical data for **(e)** root depth, **(f)** root surface area, **(g)** root growth rate for *pMtBG2:GUS* and *p35S:CaTIFY4b* transformed hairy roots of seedlings cultured on Fahraeus Plant medium plates. Plants were phenotyped for root traits at 28 days after transformation with *A. rhizogenes*. Root growth rate was calculated for the interval between 24-28 days after transformation. Error bars, S.E.; statistical significance was determined by a two-sample t-test: **P*<0.05, ***P*<0.01.

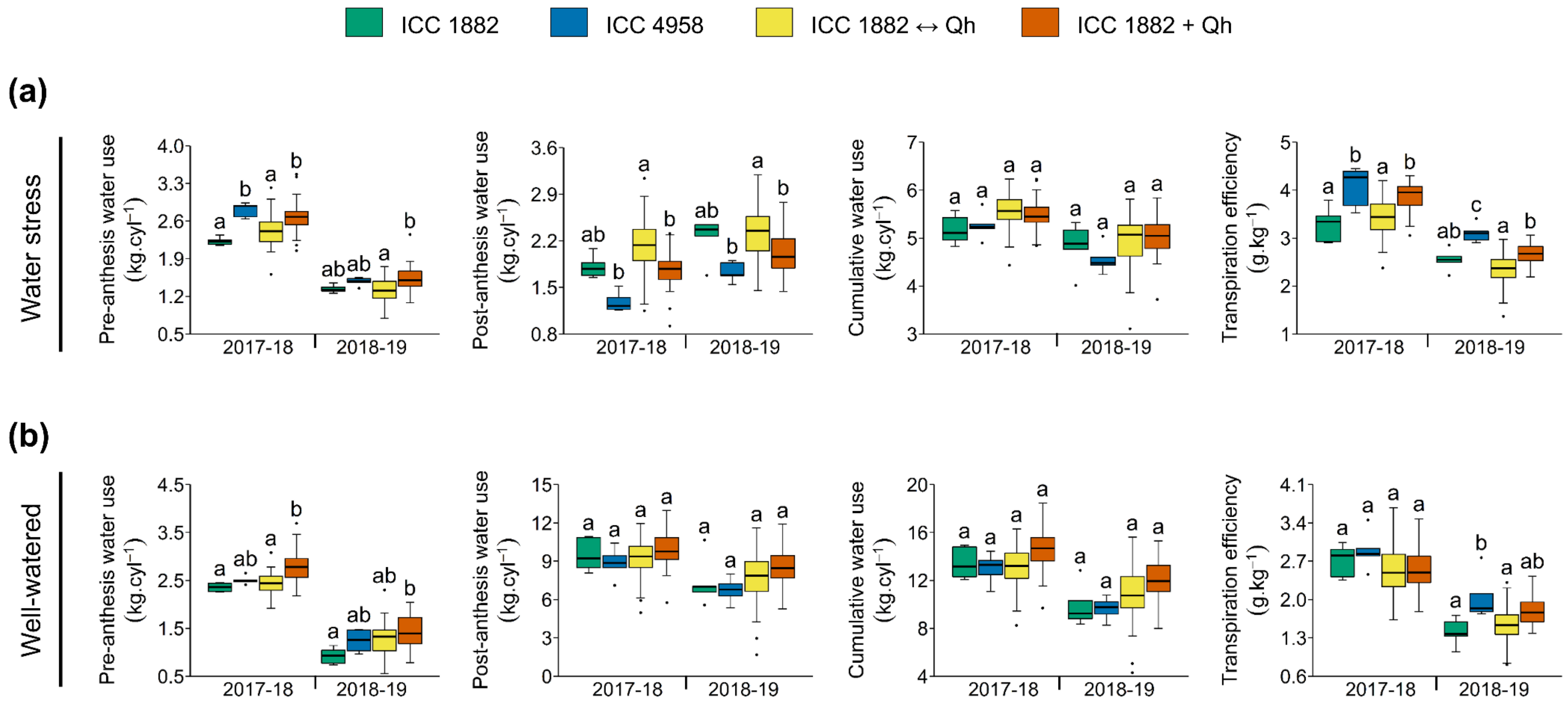

Figure S9. Effect of *"QTL-hotspot"* on water use-related traits evaluated under rain-out shelter environments. Comparison of pre-anthesis water use, post-anthesis water use, cumulative water use, and transpiration efficiency in the ICC 1882, ICC 4958, ICC 1882 ↔ Qh and ICC 1882 + Qh plants grown under **(a)** water stress and **(b)** well-watered conditions. The season in which all phenotypic traits were evaluated are shown on the x-axis. All phenotypic data were measured from plants that were grown in lysimeters under rain-out shelter conditions. For the box plots, boxes denote the 25th-75th percentile, whiskers denote the full data range and the center lines denote medians. The alphabets above the boxes (a, b, c) designate statistical significance between the groups of genotypes computed using Tukey's test ($P < 0.05$) in panels **(a,b)**.

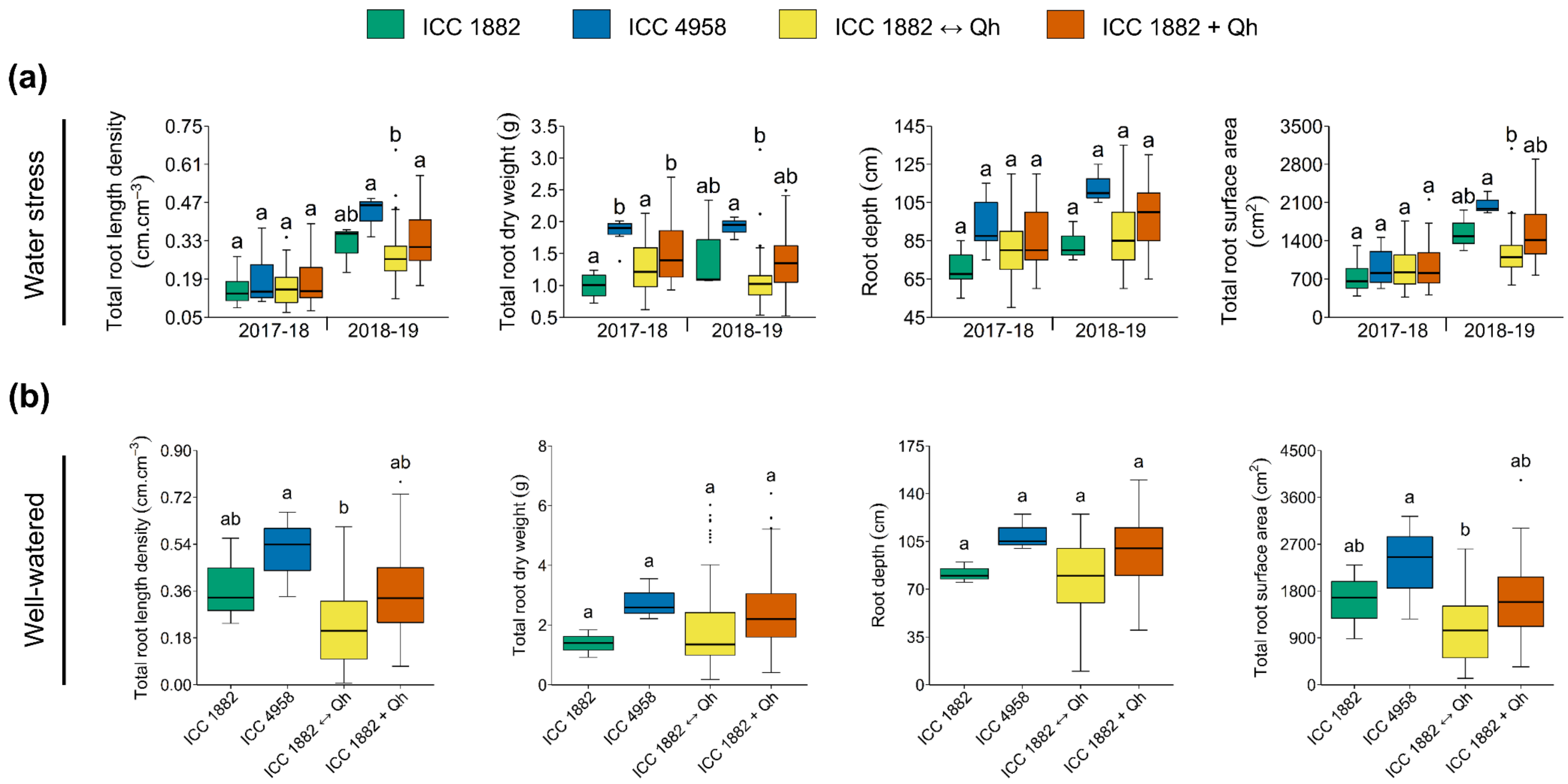

Figure S10. Phenotypic characterization of root growth and architecture traits evaluated at pod-filling stage of crop growth. Comparison of total root length density, total root dry weight, root depth, and total root surface area in the ICC 1882, ICC 4958, ICC 1882 ↔ Qh, and ICC 1882 + Qh plants grown under **(a)** water stress and **(b)** well-watered conditions. Under water stress conditions, the year in which all phenotypic traits were evaluated are shown on the x-axis. Under well-watered conditions, all root traits were evaluated only during 2018-19 crop season. For the box plots, boxes denote the 25th-75th percentile, whiskers denote the full data range and the center lines denote medians. The alphabets above the boxes (a, b) designate statistical significance between the groups of genotypes computed using Tukey's test (P<0.05) in panels **(a,b)**.

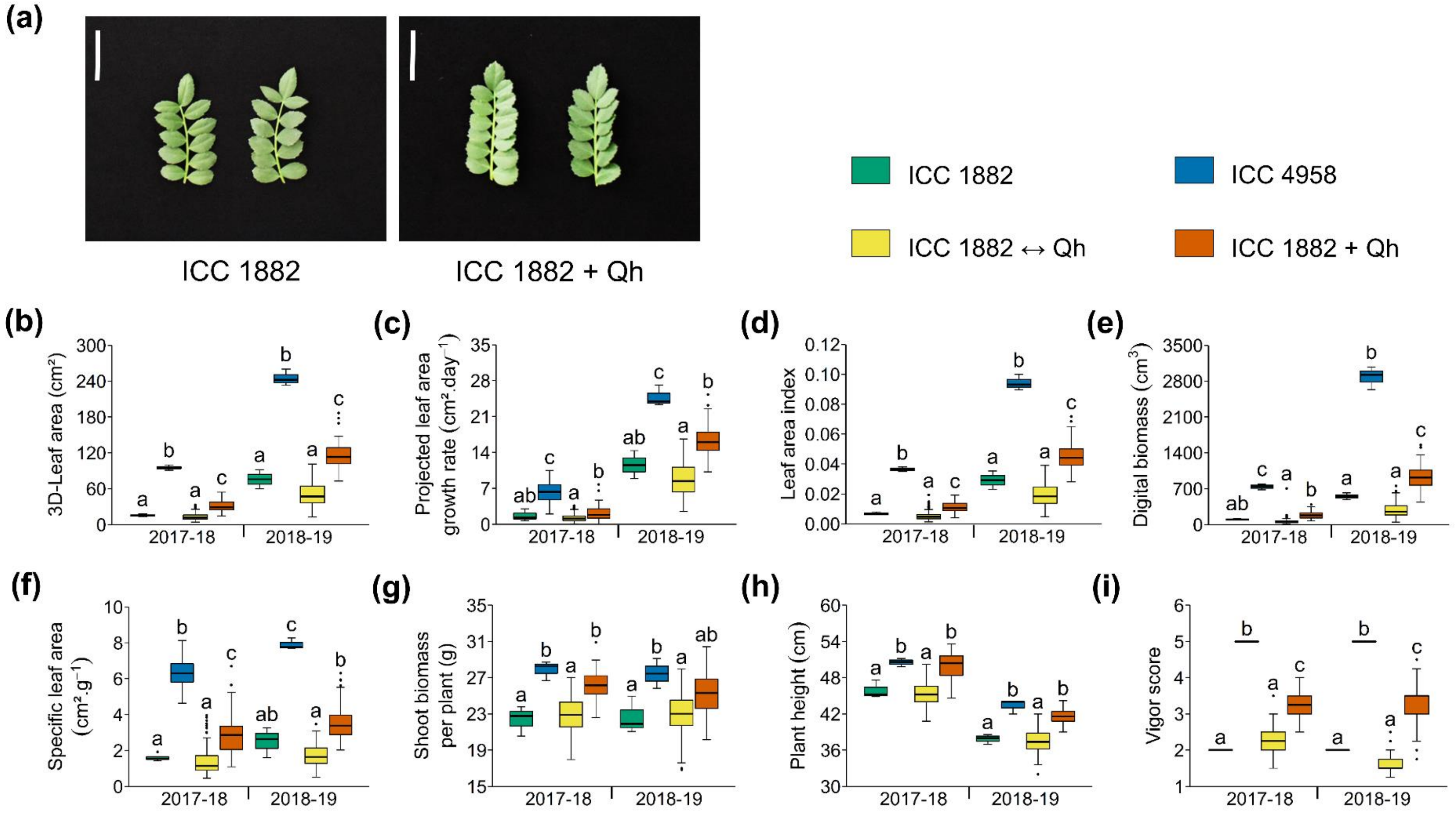

Figure S11. Effect of *"QTL-hotspot"* on canopy development traits phenotyped at LeasyScan platform and under field conditions. **(a)** Representative images of leaves of ICC 1882 and ICC 1882 + Qh plants at 20 days after sowing. Scale bars, 3 cm. **(b-i)** Comparison of **(b)** 3D-leaf area, **(c)** projected leaf area, **(d)** leaf area index, **(e)** digital biomass, **(f)** specific leaf area, **(g)** shoot biomass per plant, **(h)** plant height, and **(i)** vigour score in the ICC 1882, ICC 4958, ICC 1882 ↔ Qh, and ICC 1882 + Qh plants. The canopy development traits were evaluated at LeasyScan **(b-f)** and under field conditions **(g-i)**. The year in which all phenotypic traits were evaluated are shown on the X-axis. For the box plots, boxes denote the 25th-75th percentile, whiskers denote the full data range and the center lines denote medians. The alphabets above the boxes (a, b, c) designate statistical significance between the groups of genotypes computed using Tukey's test ($P<0.05$) in panels **(b-i)**.

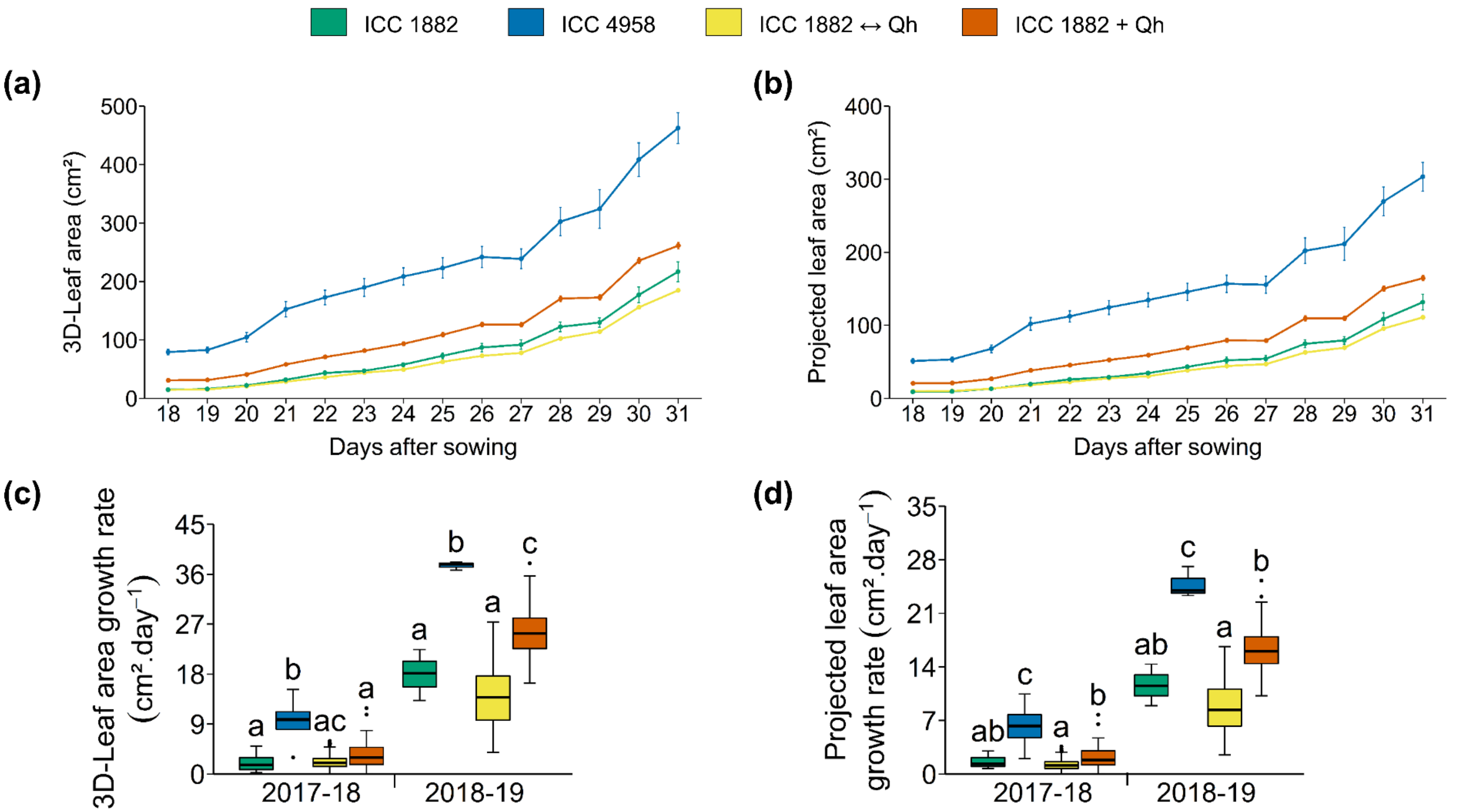

Figure S12. Time course analysis and variation in the growth rate of 3D-leaf area and projected leaf area.
Time course of **(a)** 3D-leaf area and **(b)** projected leaf area in the ICC 1882, ICC 4958, ICC 1882 ↔ Qh and ICC 1882 + Qh plants grown at LeasyScan under well-watered conditions (2017-18). Data are shown as mean ± S.E. Comparison of **(c)** 3D-leaf area growth rate and **(d)** projected leaf area growth rate in the ICC 1882, ICC 4958, ICC 1882 ↔ Qh and ICC 1882 + Qh plants. The season in which both the phenotypic traits were evaluated are shown on the x-axis. For the box plots, boxes denote the 25th-75th percentile, whiskers denote the full data range and the center lines denote medians. The alphabets above the boxes (a, b, c) designate statistical significance between the groups of genotypes computed using Tukey's test ($P < 0.05$) in panels **(c,d)**.

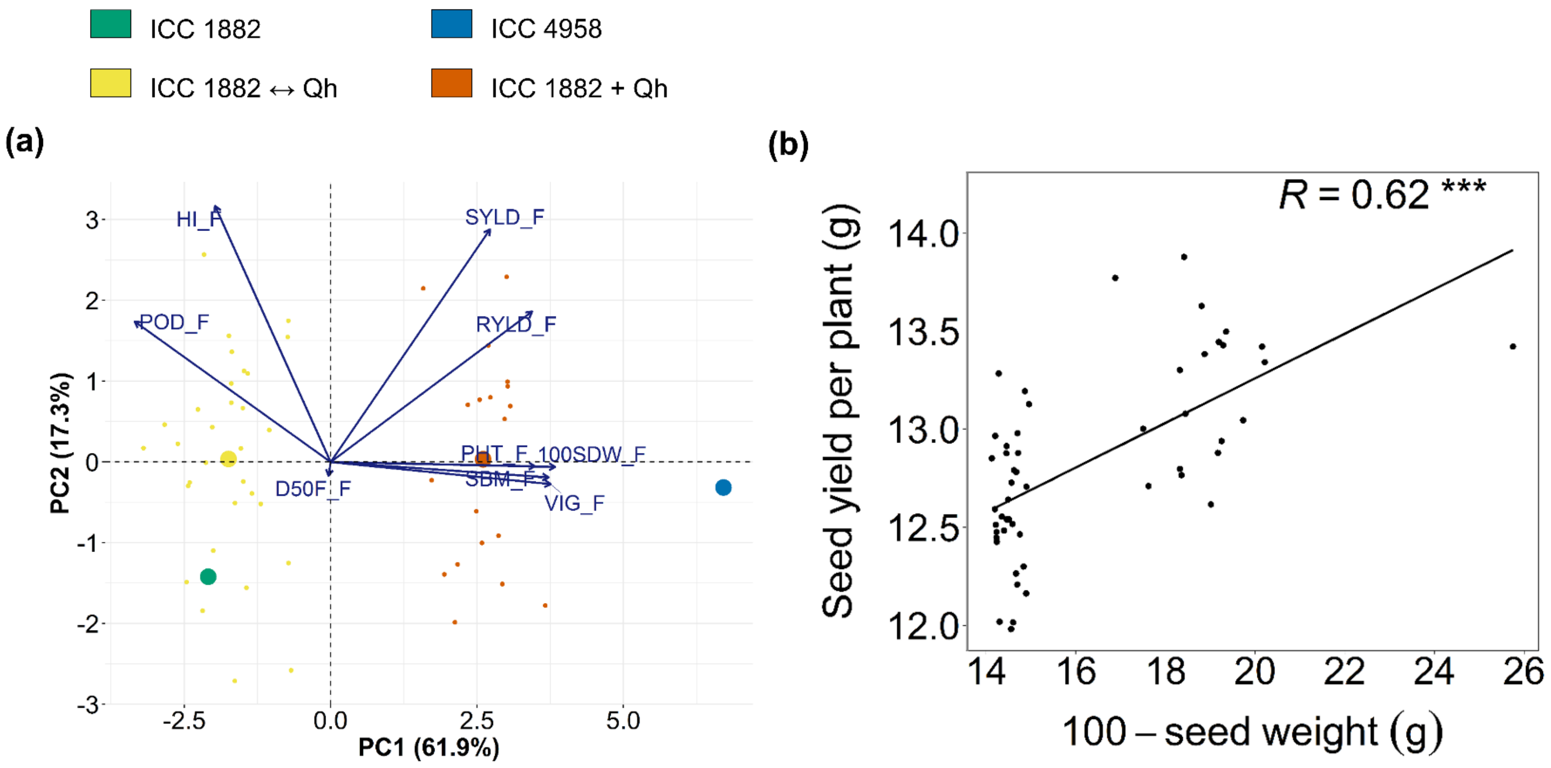

Figure S13. Principal component analysis and correlation analysis for traits measured under field conditions.
(a) Principal component analysis for traits evaluated under rainfed field conditions (2017-18). The suffix to the trait code represents the platform (F- field, R- rain-out shelter, GH- glasshouse, LS- LeasyScan) at which the traits were phenotyped. Traits are indicated by their acronyms as described in Table S5. **(b)** Relationship between seed yield per plant and 100-seed weight from field conditions (2017-18). Data represent the means of replicated plants for each genotype. R, coefficient of correlation. **, significance at $P < 0.01$; ***, significance at $P < 0.001$. In the PCA factor graph, a high correlation among the analyzed traits is represented by a similar orientation of the blue vector. The individual contribution (in percentage) to each dimension is indicated in the axis legend. Colored dots represent ICC 1882, ICC 4958, ICC 1882 ↔ Qh, and ICC 1882 + Qh plants and their position correspond to specific trait loadings relative to PC1 and PC2. Outliers in the genotype-dependent clustering of individual data sets may result due to an incorrect assignment. PC, principal component.

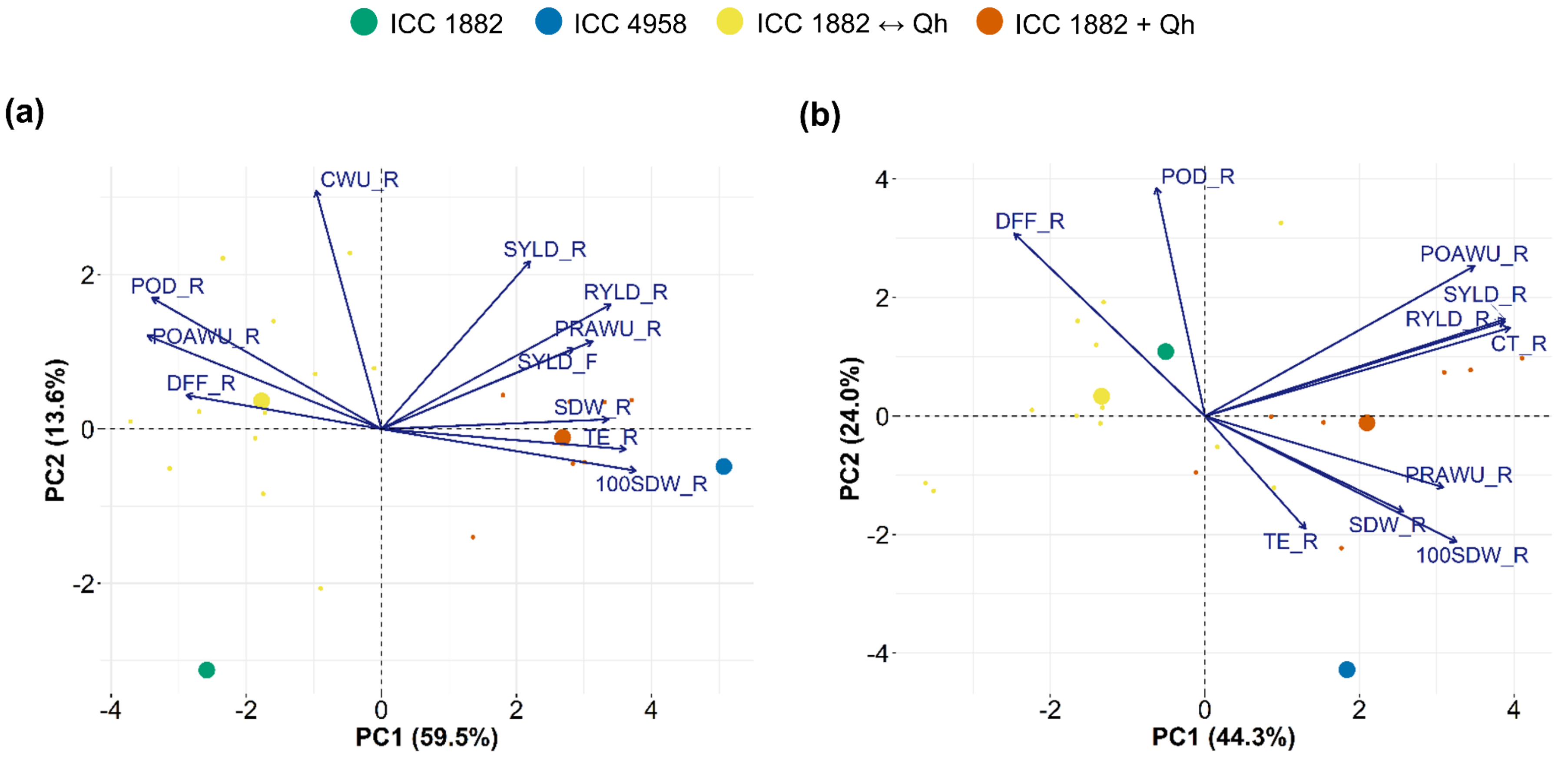

Figure S14. Principal component analysis for traits measured at rain-out shelter and field conditions.
(a) Principal component analysis for seed yield from field and traits from rain-out shelter lysimeters (2017-18 WS). **(b)** Principal component analysis for traits from rain-out shelter lysimeters under well-watered conditions (2017-18 WW). The suffix to the trait code represents the platform (F- field, R- rain-out shelter, GH- glasshouse, LS- LeasyScan) at which the traits were phenotyped. Traits are indicated by their acronyms as described in Table S5. A high correlation among the analyzed traits is represented by a similar orientation of the blue vector. The individual contribution (in percentage) to each dimension is indicated in the axis legend. Colored dots represent ICC 1882, ICC 4958, ICC 1882 ↔ Qh, and ICC 1882 + Qh plants and their positions correspond to specific trait loadings relative to PC1 and PC2. Outliers in the genotype-dependent clustering of individual data sets may result due to an incorrect assignment. PC, principal component.

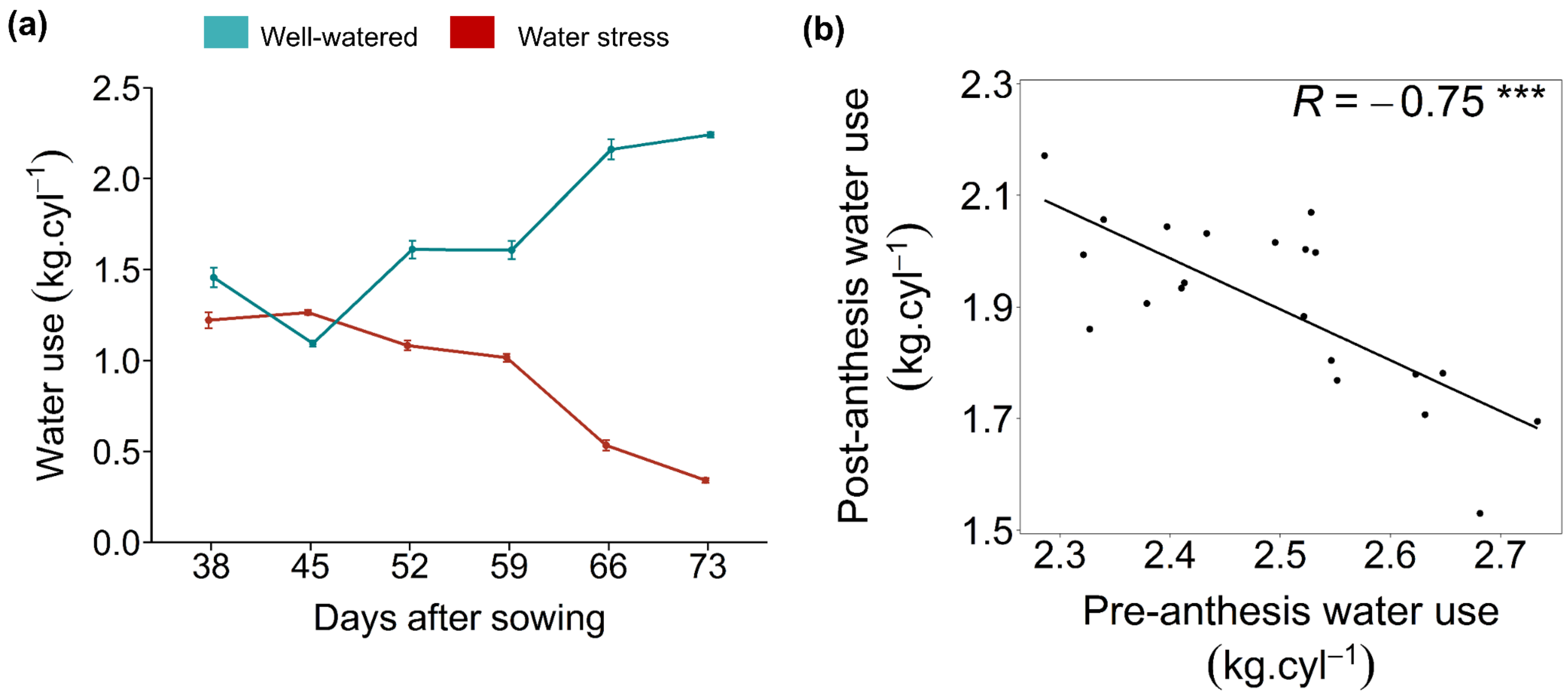

Figure S15. Time course analysis of water uptake profile and relationship between water uptake in pre-anthesis and post-anthesis stage. **(a)** Time course of total water used (kg cyl^{-1}) in the ICC 1882, ICC 4958, ICC 1882 ↔ Qh, and ICC 1882 + Qh plants grown in lysimeters under well-watered and water stress conditions at rain-out shelter (2017-18). Data are shown as mean ± S.E. **(b)** Relationship between water used (kg cyl^{-1}) in the pre-anthesis and post-anthesis period for chickpea genotypes grown in 1.2 m length lysimeters under water stress conditions at rain-out shelter (2017-18 WS). Data indicate the means of five replicated plants per genotype and treatment. ***, significance at $P < 0.001$.

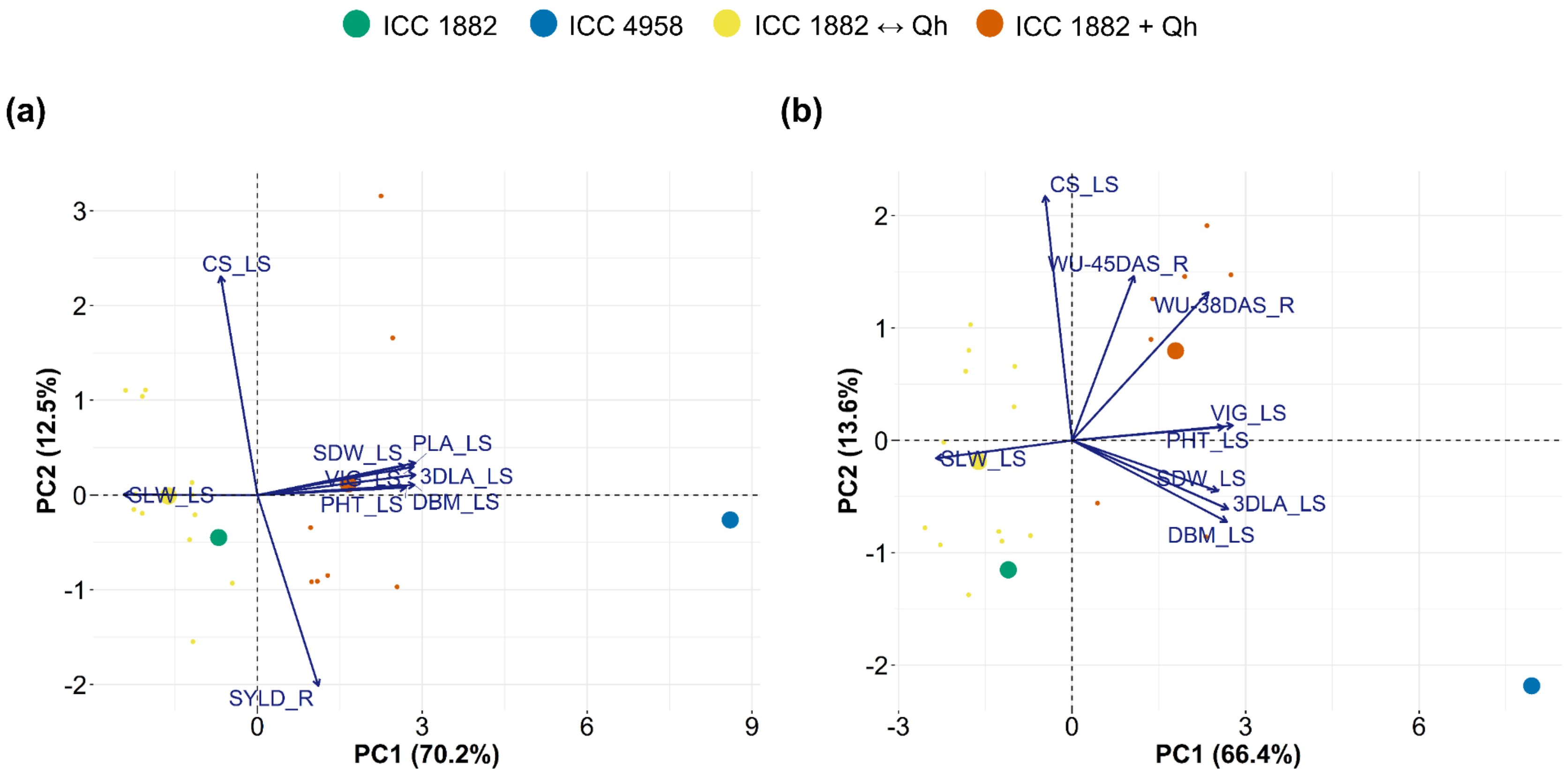

Figure S16. Principal component analysis for phenotypic traits evaluated at LeasyScan and rain-out shelter.
(a) Principal component analysis for seed yield evaluated at rain-out shelter (2017-18 WW) and canopy development traits from LeasyScan (2017-18). **(b)** Principal component analysis for early water extraction (WU-38DAS and WU-45DAS) traits from rain-out shelter lysimeters (2017-18 WS) and canopy development traits from LeasyScan (2017-18). The suffix to the trait code represents the platform (F- field, R- rain-out shelter, GH- glasshouse, LS- LeasyScan) at which the traits were phenotyped. Traits are indicated by their acronyms as described in Table S5. A high correlation among the analyzed traits is represented by a similar orientation of the blue vector. The individual contribution (in percentage) to each dimension is indicated in the axis legend. Colored dots represent ICC 1882, ICC 4958, ICC 1882 ↔ Qh, and ICC 1882 + Qh plants and their position correspond to specific trait loadings relative to PC1 and PC2. Outliers in the genotype-dependent clustering of individual data sets may result due to an incorrect assignment. PC, principal component.

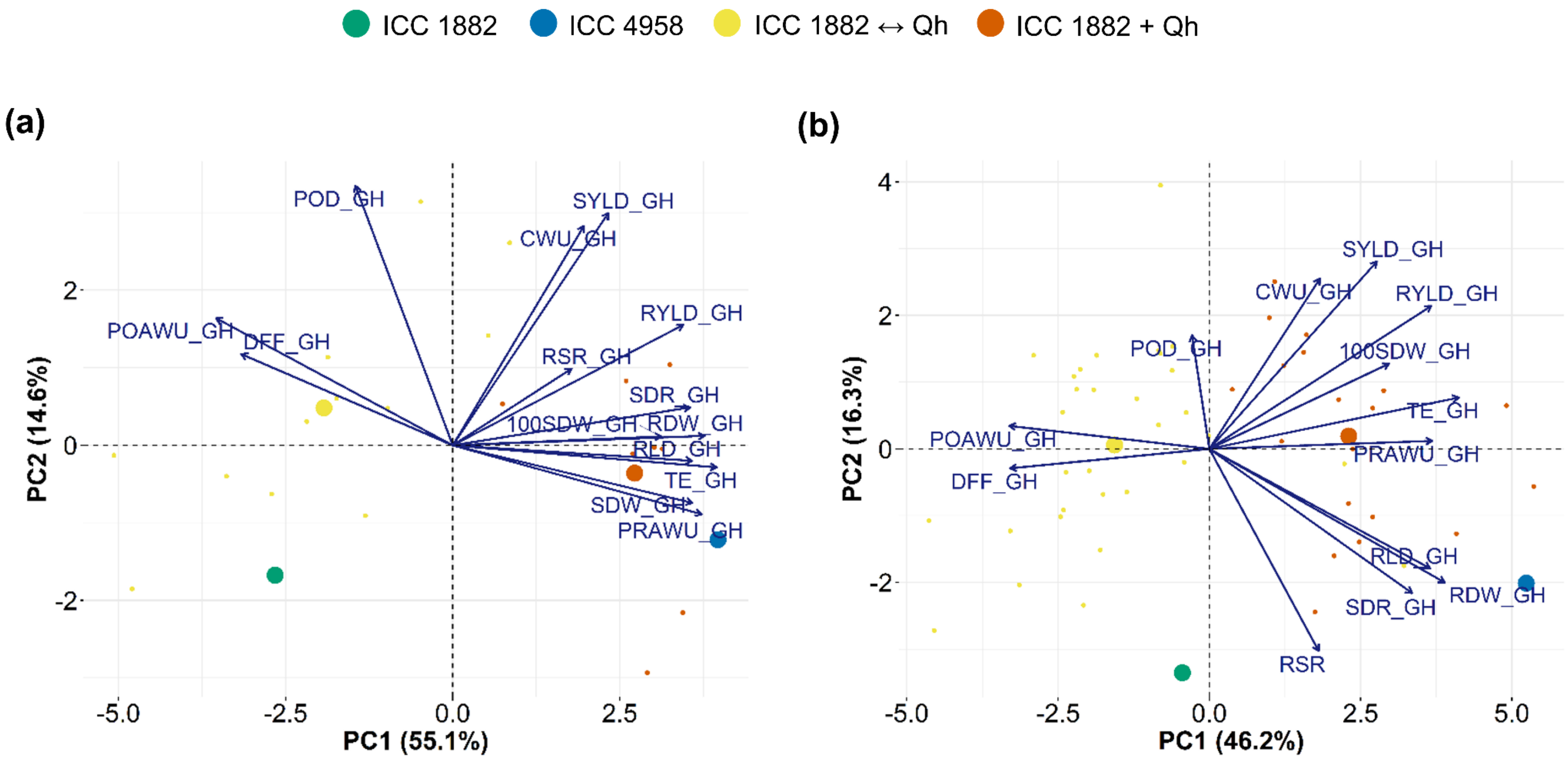

Figure S17. Principal component analysis for phenotypic traits evaluated under glasshouse environments.
(a) Principal component analysis for root traits measured at vegetative stage and traits from glasshouse lysimeters (2018-19 WS). **(b)** Principal component analysis for root, water use, and agronomic traits evaluated under water stress conditions in glasshouse (2018-19 WS). The suffix to the trait code represents the platform (F- field, R- rain-out shelter, GH- glasshouse, LS- LeasyScan) at which the traits were phenotyped. Traits are indicated by their acronyms as described in Table S5. A high correlation among the analyzed traits is represented by a similar orientation of the blue vector. The individual contribution (in percentage) to each dimension is indicated in the axis legend. Colored dots represent ICC 1882, ICC 4958, ICC 1882 ↔ Qh, and ICC 1882 + Qh plants and their position correspond to specific trait loadings relative to PC1 and PC2. Outliers in the genotype-dependent clustering of individual data sets may result due to an incorrect assignment. PC, principal component.

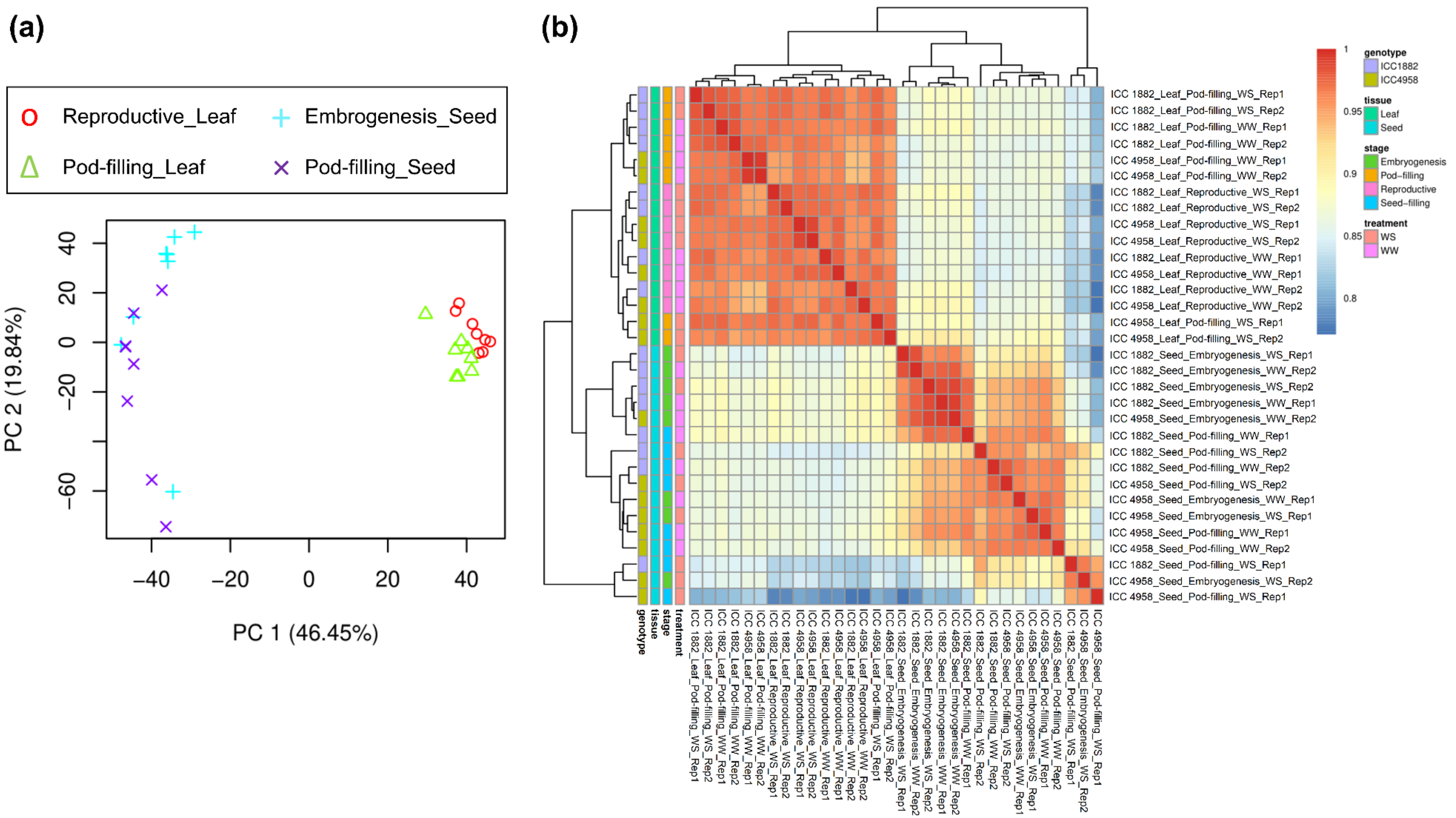

Figure S18. Principal component analysis and correlation analysis between seed and leaf transcriptomes of parental genotypes at different development stages and water treatments.

(a) Principal component analysis plot displaying clustering of leaf and seed transcriptomes at different development stages in ICC 4958 and ICC 1882. **(b)** Heatmap showing hierarchical clustering of Pearson correlation for 16 seed and leaf samples included in the analysis. Color scale indicates the degree of correlation. Clustering of samples were based on the pair-wise correlations.

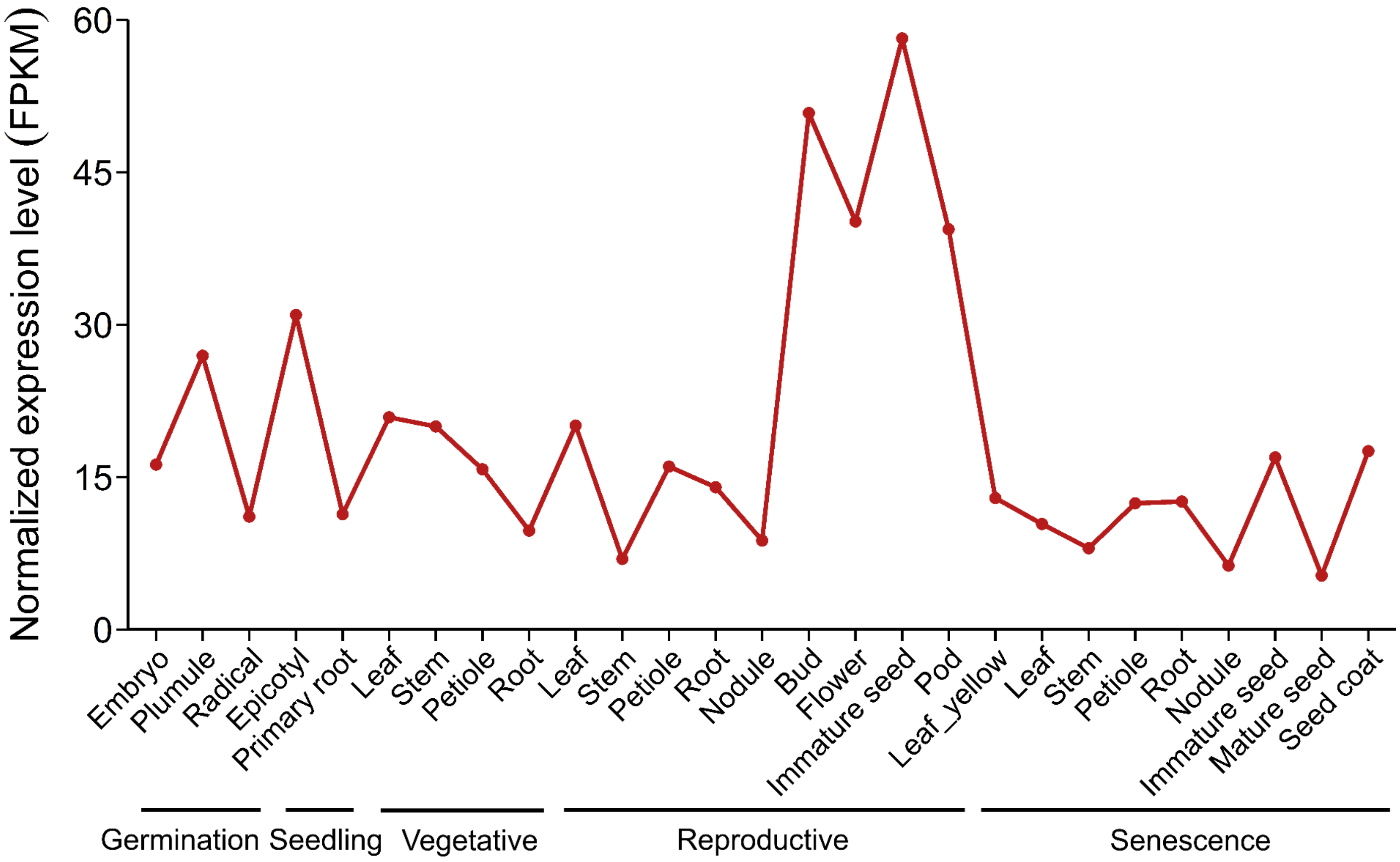

Figure S19. *In silico* analysis of *CaTIFY4b* gene expression.
Expression of *CaTIFY4b* gene in different tissues as shown by *Cicer arietinum* Gene Expression Atlas. The *CaTIFY4b* gene shows highest expression in immature seed at the reproductive stage.

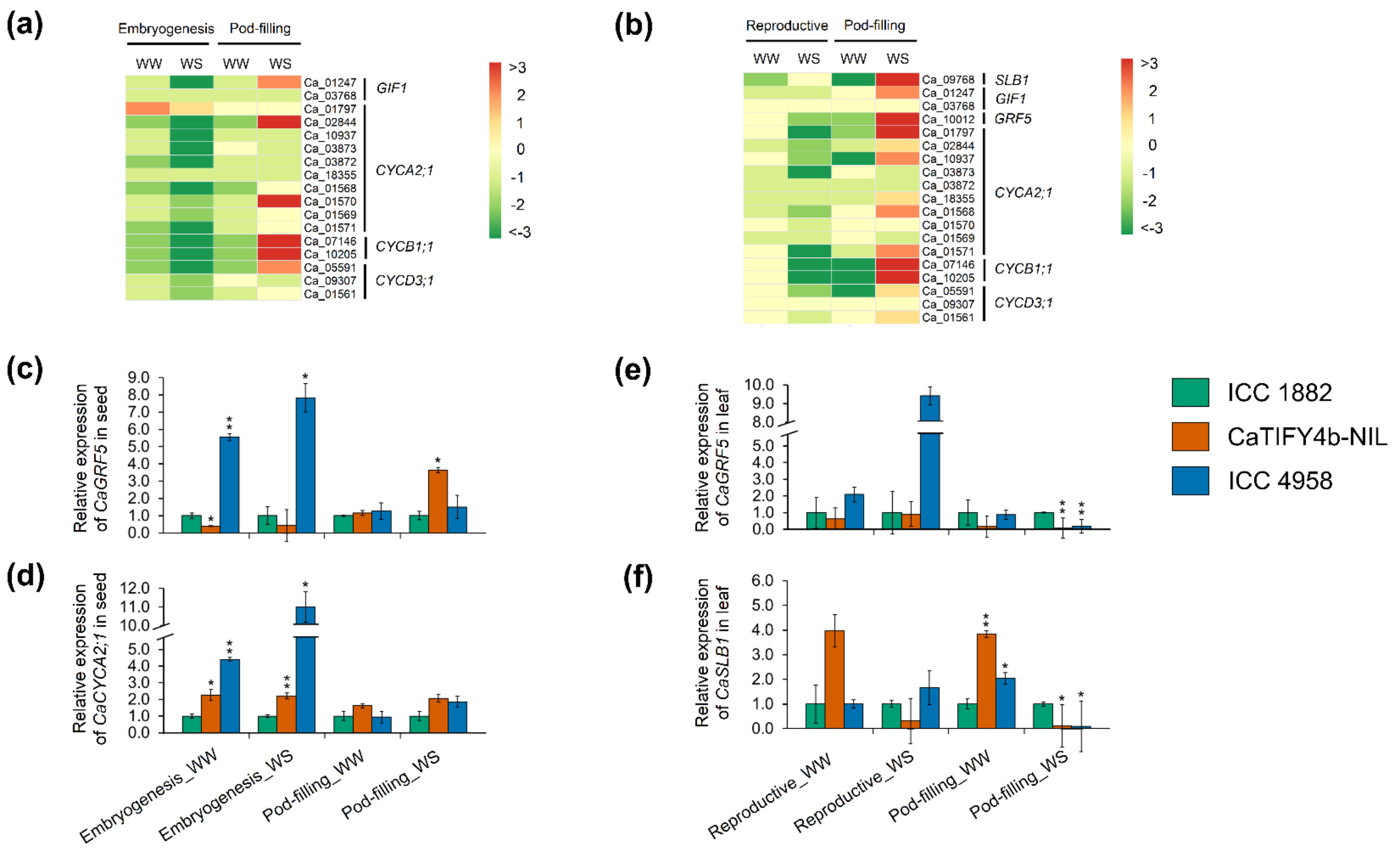

Figure S20. Transcriptome and quantitative RT-PCR analysis of CaTIFY4b downstream target genes in seed and leaf samples. Heatmaps showing expression profiles of genes identified as downstream target genes of *CaTIFY4b* orthologs in other plant species in **(a)** seed and **(b)** leaf samples at different development stages, under well-watered (WW) and water stress (WS) conditions. The color scale represents log2 fold change. The relative expression levels of **(c)** *CaGRF5* and **(d)** *CaCYCA2;1* genes at the embryogenesis and pod-filling stage under WW and WS conditions, in the seeds of ICC 1882, CaTIFY4b-NIL, and ICC 4958 were detected by qRT-PCR (n=3). The relative expression levels of **(e)** CaGRF5 and **(f)** *CaSLB1* genes at the reproductive and pod-filling stage under WW and WS conditions, in the leaves of ICC 1882, CaTIFY4b-NIL, and ICC 4958 were detected by qRT-PCR (n=3). Data was normalized with *GAPDH*. *P<0.05, Student's t-test.
